# Extracellular vesicles from gastric epithelial GES-1 cells infected with *Helicobacter pylori* promote changes in recipient cells associated with malignancy

**DOI:** 10.3389/fonc.2022.962920

**Published:** 2022-10-12

**Authors:** María Fernanda González, Renato Burgos-Ravanal, Baohai Shao, Jay Heinecke, Manuel Valenzuela-Valderrama, Alejandro H. Corvalán, Andrew F. G. Quest

**Affiliations:** ^1^ Laboratorio de Comunicaciones Celulares, Centro de Estudios en Ejercicio, Metabolismo y Cáncer (CEMC), Programa de Biología Celular y Molecular, Facultad de Medicina, Universidad de Chile, Santiago, Chile; ^2^ Facultad de Ciencias Químicas y Farmacéuticas, Universidad de Chile, Centro Avanzado para Estudios en Enfermedades Crónicas (ACCDIS), Santiago, Chile; ^3^ Division of Metabolism, Endocrinology and Nutrition, University of Washington, Seattle, WA, United States; ^4^ Laboratorio de Microbiología Celular, Instituto de Investigación y Postgrado, Universidad Central de Chile, Santiago, Chile; ^5^ Departamento de Hematología-Oncología, Facultad de Medicine, Pontificia Universidad Católica de Chile, Santiago, Chile

**Keywords:** extracellular vesicles, *Helicobacter pylori*, gastric cancer, exosomes, malignancy

## Abstract

Chronic *Helicobacter pylori* (*H. pylori*) infection is considered the main risk factor for the development of gastric cancer. Pathophysiological changes in the gastric mucosa initiated by this bacterium can persist even after pharmacological eradication and are likely attributable also to changes induced in non-infected cells as a consequence of intercellular communication *via* extracellular vesicles (EVs). To better understand what such changes might entail, we isolated EVs from immortalized normal gastric GES-1 cells infected (EVHp+) or not with *H. pylori* (EVHp-) by ultracentrifugation and characterized them. Infection of GES-1 cells with *H. pylori* significantly increased the release of EVs and slightly decreased the EV mean size. Incubation with EVHp+ for 24 h decreased the viability of GES-1 cells, but increased the levels of IL-23 in GES-1 cells, as well as the migration of GES-1 and gastric cancer AGS cells. Furthermore, incubation of GES-1 and AGS cells with EVHp+, but not with EVHp-, promoted cell invasion and trans-endothelial migration *in vitro*. Moreover, stimulation of endothelial EA.hy926 cells for 16 h with EVHp+ promoted the formation of linked networks. Finally, analysis by mass spectrometry identified proteins uniquely present and others enriched in EVHp+ compared to EVHp-, several of which are known targets of hypoxia induced factor-1α (HIF-1α) that may promote the acquisition of traits important for the genesis/progression of gastric pre-neoplastic changes associated with *H. pylori* infection. In conclusion, the harmful effects of *H. pylori* infection associated with the development of gastric malignancies may spread *via* EVs to non-infected areas in the early and later stages of gastric carcinogenesis.

## Introduction

Gastric cancer (GC) is the fourth leading cause of cancer-related mortality and the fifth most common cancer worldwide (1). Infection with bacterial and viral agents promotes cancer in different epithelia, in part due to persistent inflammation during infection (2). Accordingly, infection with the gram-negative bacteria *Helicobacter pylori* (*H. pylori*) is considered the main risk factor for the development of GC (3–9). *H. pylori* colonizes the gastric epithelium of 50.8% of the population in developing countries compared to 34.7% in developed countries (10), and is directly related to the development of GC (11). In particular, GC induced by *H. pylori* has been associated with chronic inflammation, characterized by the accumulation of proinflammatory cytokines, such as TNF-α, IL-1β, IL-6, and IL-8 (12). Another less mentioned interleukin, but which has recently been described as important in the context of *H. pylori* infection and development of gastric cancer, is IL-23 (13, 14). The expression of IL-23 is increased in patients with *H. pylori* infection and its levels positively correlate with the degree of neutrophil and monocyte infiltration (15). One study even reports that the serum concentrations of IL-23A could represent a biomarker for poor clinical prognosis in patients with GC (16). Moreover, a study in patients revealed that specific polymorphisms in the gene encoding for IL-23 receptor (IL-23R) were associated with reduced GC survival rates (14).

Infection with *H. pylori* causes gastric tissue lesions that are triggered by initial gastric cell death (17). The remaining gastric cell population shows adaptive responses, related to cell survival and proliferation (17). These adaptive responses also include increased cell migration (18–20). The acquisition of these malignant features may facilitate the progression of gastric precancerous lesions. Early clinical follow-up studies indicated that treatment with antibiotics to eradicate *H. pylori* in patients reduces the progression of preneoplastic lesions (21). Moreover, a meta-analysis by Hai-Ning Chen et al. (2016) suggests that the eradication of *H. pylori* can reduce the incidence of GC, particularly in patients diagnosed at a stage prior to intestinal metaplasia (IM). However, when the diagnosis is IM or dysplasia, no preventive effect was observed after eradication, neither in the risk of developing GC nor in the progression to a precancerous lesion (22). These observations suggest that irreversible changes occurred at this stage leading to the development of GC.

Extracellular vesicles (EVs) are cell-derived vesicles secreted by virtually all eukaryotic and prokaryotic cells that carry proteins, nucleic acids and lipids derived from the parent cell. Once these EVs reach a recipient cell, they induce changes according to the profile of molecules they contain (23). Intercellular communication through EVs is of importance in different physiological and pathophysiological events (24). The pathophysiological processes include inflammation, cell proliferation, angiogenesis, and migration (24). The role of EVs in inflammation is linked to their ability to transport cytokines and other immune-related molecules that can contribute to the spread of inflammation (25). Growing evidence indicates that EVs also play a crucial role in pathogen-related diseases, as they can transport pathogen-derived molecules, in addition to host-derived molecules acquired during infection (26). Moreover, in GC, EVs have the potential to promote malignant changes at all stages of cancer development (27).

Currently, little is known about the role of EVs released by host-cells during *H. pylori* infection. Moreover, to our knowledge, no studies have fully characterized EVs released from gastric cells after infection with *H. pylori*. Here we characterized the EVs released from non-tumorigenic gastric epithelial GES-1 cells after *H. pylori* infection. Furthermore, we determined whether these EVs can perpetuate the cellular changes initiated by *H. pylori* infection, in the absence of the pathogen. We observed that infection with *H. pylori* caused changes in the release of host derived EVs. Furthermore, we obtained evidence suggesting that EVs released from host-cells after *H. pylori* infection induced biological changes in recipient gastric and endothelial cells, possibly mediated by proteins relating in function to cell adhesion/migration, which could favor the development of GC.

## Materials and methods

### Cell culture

The immortalized human gastric epithelium cell line GES-1 (kindly provided by Dr. Armando Rojas, Universidad Católica del Maule, Chile) and the gastric adenocarcinoma cell line AGS (ATCC^®^ CRL-1793™) were cultured in RPMI-1640 medium (Gibco, Thermo Fisher Scientific, Waltham, Massachusetts, USA). The gastric cancer cell line Hs746T (ATCC^®^ HTB-135™) was cultured in DMEM High glucose medium (Gibco, Thermo Fisher Scientific, Waltham, Massachusetts, USA). EA.hy926 endothelial cells (kindly provided by Dr. Gareth Owen, Pontificia Universidad Catolica de Chile, Chile) were cultured in IMDM medium (Gibco, Thermo Fisher Scientific, Waltham, Massachusetts, USA). All media were supplemented with 10% fetal bovine serum (FBS, European Grade, Heat Inactivated, Biological Industries, Israel) and antibiotics (10,000 U/ml penicillin and 10 μg/ml streptomycin), unless other conditions are specified. Cells were cultured in a controlled atmosphere (5% CO_2_, 70% humidity) at 37°C.

### Bacterial culture

The *H. pylori* strain 26695 was obtained from the ATCC (American Type Culture Collection, 700392). The bacteria were grown on trypticase soy agar (TSA) plates, supplemented with 5% horse donor serum (Donor Horse Serum, Biological Industries, Israel), the nutrient supplement Vitox (2% Oxoid Limited, Wade Road, Basingstoke, Hampshire, UK) and the selective supplement Dent (0,2% Oxoid Limited) for 24 h under low oxygen conditions (5% CO_2_, 70% humidity) at 37°C.

### Obtaining the conditioned medium from GES-1 cells infected with *H. pylori* to isolate EVs from GES-1 cells infected with *H. pylori* (EVHp+)

GES-1 cells were seeded onto 875cm² 5-layer Multi-Flasks (#353144, Falcon, Life Sciences, Durham NC, USA) and cultured for 24 h. For this purpose the medium RPMI-1640, for this purpose the medium RPMI-1640 (Gibco) supplemented with 10% FBS was used without antibiotics. For infection, bacteria were collected in PBS, centrifuged at 4,000 x *g* and resuspended in PBS. An absorbance of 0.4 units at 560 nm was considered equivalent to 3x10^8^ bacteria. Cells were infected with a multiplicity of infection (MOI) of 1:100, as previously described (28). After 24 h post-infection, bacteria were eliminated by washing the cells five times with PBS and incubating them with gentamycin (final concentration 200 μg/ml) in RMPI medium for 1 h. Then the cells were washed again five times with PBS and the medium was replaced with RMPI containing gentamycin (final concentration 25 μg/ml) supplemented with 5% EV-depleted SFB (to minimize co-purification of EVs from FBS). After 48 h of incubation, conditioned media from cells infected with *H. pylori* was collected to purify EVs from GES-1 cells infected with *H. pylori* (EVHp+). In parallel, the same procedure was carried out with GES-1 cells, omitting the step of adding the bacteria, to collect the conditioned medium to purify EVs of GES-1 cells that had not been infected with *H. pylori* (EVHp-). At the time of collecting the conditioned medium, the cells were counted by trypan blue staining in order to subsequently estimate the number of EVs released per cell. EV-depleted FBS was prepared by centrifuging the FBS at 100,000 × *g* for 18 h at 4°C using a T-1250 rotor (Sorvall WX+100, Thermo Fisher Scientific, Waltham, Massachusetts, USA) and filtering the supernatant with a 0.22 μm filter.

### Extracellular vesicle isolation

Extracellular vesicles were isolated from the culture medium of GES-1 cells, infected or not with *H. pylori*, by differential centrifugation, using protocols similar to those described in other studies for gastric cells (29–31). Approximately 200 ml of conditioned media were obtained for each condition. Following these protocols, the conditioned media were subjected to an initial centrifugation step at low speed (2,000 × *g*) for 30 min (Universal 320R Centrifuge, Hettich, Sigma-Aldrich, Beverly, Massachusetts, USA). The supernatant was filtered through 0.22 μm filters and then centrifuged at 100,000 x *g* for 80 min using a fixed angle T-1250 rotor. The resulting supernatant was discarded and the pellet was resuspended in 20 ml of PBS (2 ml per tube). The resuspended pellet was centrifuged at 100,000 x *g* for 80 min again, using the swinging bucket TH-641 rotor. EVs in the pellet were resuspended in 200 μl PBS and stored at -80°C until further analysis. The PBS used for this protocol was filtered through 0.1 mm filters.

### Determination of EV size and concentration by nanoparticle tracking analysis (NTA)

The size and concentration of EVs were determined by NTA (32). This analysis uses video recordings of the Brownian motion to determine particle size. 20 μl of every sample were diluted 1:50 in PBS (1 ml final volume) to obtain 10–100 particles per frame. This final suspension was injected into the NanoSight NS300 (Malvern Panalytical). For each sample, three videos of 30 s duration were recorded. Then, the videos were analyzed using the software Nanosight NTA 3.2 to obtain EV size and concentration.

### Western blot analysis

The presence of exosomal markers in EVs was evaluated by Western blotting. GES-1 cells and isolated EVs were lysed by sonication in lysis buffer (PBS, 0.1% SDS, and protease inhibitors: 12.5 μg/ml leupeptin, 10 μg/ml antipain, 100 μg/ml benzamidine, 1 mM phenylmethylsulphonyl fluoride, 1 mM sodium orthovanadate, and 10 mM sodium fluoride). Total protein concentration in lysate samples was determined using the BCA Protein Assay Kit (Thermo Fisher Scientific, Waltham, Massachusetts, USA) following the manufacturer’s instructions. 30 μg of each sample were loaded per well in polyacrylamide gels (3% polyacrylamide stacking gel, 10% acrylamide resolving gel) and transferred to nitrocellulose membranes, as described previously (18). Membranes were blocked with PBS containing 0.1% Tween and 5% skimmed milk for 1 h on a shaker at room temperature. The blocked membranes were incubated overnight with primary antibodies diluted in the blocking buffer at 4°C. The primary antibody dilutions were: anti-Alix (sc-53540, Santa Cruz; 1/3000); Anti-tsg101 (sc-7964, Santa Cruz; 1/250); anti-Calregulin (sc-373863, Santa Cruz; 1/500); anti-CD81 (sc-166029, Santa Cruz; 1/1000); anti-CagA (sc-28368, Santa Cruz; 1/1000); anti-Helicobacter pylori (IS523, Dako; 1/1000); anti-JAK (sc-1677, Santa Cruz; 1/1000); anti-SQSTM1 (sc-28359, Santa Cruz; 1/1000); anti-Integrin β1 (sc-8978, Santa Cruz; 1/500); anti-RhoA (sc-418, Santa Cruz; 1/500); anti-ICAM-1 (sc-8439, Santa Cruz; 1/500); anti-Syntenin-1 (133003, Synaptic Systems; 1/500) and anti-Rab5 (sc-46692, Santa Cruz; 1/500). Then, the membrane was washed and incubated with anti-mouse IgG (H&L) peroxidase-conjugated secondary antibody Rockland (#610-4302) 1/5000, or anti-rabbit IgG (H&L) peroxidase-conjugated secondary antibody (Rockland #611-1302) 1/5000. Blots were detected with SuperSignal™ West Femto (#34094, Thermo Fisher Scientific, Waltham, Massachusetts, USA).

### Determination of relative gene expression by RT-qPCR

GES-1 cells (6x10^5^) were incubated with 24 μg of EVHp+ or 24μg of EVHp-. Total RNA from cells after treatments was isolated using TRIzol (Thermo Fisher Scientific, Waltham, Massachusetts, USA) according to the manufacturer’s instructions. RNA was precipitated with isopropanol 50%, resuspended in nuclease-free water and treated with DNase (Promega, Madison, Wisconsin, USA) according to the manufacturer’s protocol to obtain DNA-free RNA. cDNA was obtained by reverse transcription (RT) using 1 μg of DNA-free RNA and reverse transcriptase enzyme M-MLV (Promega, Madison, Wisconsin, USA) with 2 μM of random primers. 2 μl of cDNA solution were used to quantify the relative gene expression. qPCRs were performed using a Brilliant II SYBR Green qPCR Master Mix (Agilent, Santa Clara, California, USA) following the manufacturer´s instructions. 20 μl reaction volumes were analyzed in the Mx3000P QPCR System (Agilent, Santa Clara, California, USA) under the following conditions: initial 10 min denaturation at 95°C, followed by 40 cycles at 95°C for 20 s, annealing temperature for 20 s and at 72°C for 20 s. The primer sequences and annealing temperatures used for qPCRs analysis are presented in **Supplementary Table 1**. Relative gene expression levels were calculated by the 2-(ΔΔCT) method (33) using 18S rRNA as an extrinsic internal control.

### Cell viability

Cell viability was evaluated using the trypan blue dye exclusion assay. 3x10^5^ cells were stimulated with 12 μg of EVHp- or EVHp+ for 24 h. Then, cell suspensions were stained with 0.4% trypan blue solution and viable cells were counted in a Neubauer chamber (34).

### Migration and invasion assays

The effects on GES-1, AGS and Hs746T cell migration following treatment with EVs were evaluated in Boyden Chambers (Transwell Costar, 6.5 mm diameter, 8 μm pore size; Corning, Kennebunk, Maine, USA), whereas invasion was evaluated in Corning^®^ BioCoat^®^ Matrigel^®^ Invasion Chambers (Bedford, Massachusetts, USA). Cells (3x10^5^) were treated with 12 μg EVHp- or EVHp+ for 24 h, washed and resuspended in serum-free medium, then counted and added to the top of each chamber insert. The bottom sides of the transwell inserts were coated with 2 μg/ml fibronectin. Medium supplemented with FBS was added to the bottom chamber. After 2 h for GES-1, 5 h for AGS cells and 16 h for Hs746T, transwell inserts were removed, and cells were stained with 0.1% crystal violet in 2% ethanol. For invasion, GES-1 and AGS cells were allowed to invade for 18 h and Hs746T cells were allowed to invade for 20 h. Subsequently, cells were stained and fixed using toluidine blue and fixation solution (4% paraformaldehyde in 100 mM PIPES buffer, pH 6.8, containing 0.04 M KOH, 2 mM EGTA, and 2 mM MgCl2). Stained cells that migrated and invaded toward the lower side of the insert were washed, photographed (Oxion Inverso biological microscopes, Euromex, microscopes Holland) and counted.

### Transendothelial migration assay

Endothelial EA.hy926 cells (2x10^5^) were seeded in Boyden Chambers (Transwell Costar, 6.5 mm diameter, 8 µm pore size) and cultured for 72 h to allow the formation of an impermeable cell monolayer. The permeability of this layer was corroborated by adding dextran blue dye (Sigma-Aldrich) to the upper chamber and evaluating the appearance of dye in the lower chamber of the transwell. Once the endothelial cell monolayer was confirmed as being impermeable to dye, the GES-1 or AGS cells that were stimulated with EVs (as in the section 2.8), were stained with CellTracker Green (Thermo Fisher Scientific, Waltham, Massachusetts, USA) counted and added on top of the endothelial monolayer. After 18 h of incubation, the assay was stopped by fixing the cells in fixation solution for 15 min and washing the transwells 2 times for 5 min with universal buffer (0.15 N NaCl, 50 mM Tris-HCl pH 7.5, 0.1% sodium azide). The images from the fluorescent cells that transmigrated were taken using a fluorescence microscope (Spinning disk Olympus IX81) (Tokyo, Japan).

### *In vitro* vascular network formation assay

The formation of linked networks of endothelial EA.hy926 cells following EV treatment was evaluated as follows: First, EA.hy926 cells were deprived of serum for at least 4 h before initiating the experiment. Then, 50 µl of matrigel per well were added to polylysine-coated 96well plates and incubated at 37°C for at least 1 h. EA.hy926 cells were then seeded onto the wells and 10 μg of EVHp- or EVHp+ were added. VEGF was used as a positive control. After 16 h, photographs were taken (Oxion Inverso biological microscopes, Euromex, microscopes Holland) and the formation of linked networks was determined by using the ImageJ software to measure the master segments.

### Analysis of EV proteome by mass spectrometry

Shotgun proteomics analyses were performed in positive ion mode with an ultra-high mass resolution Orbitrap Fusion Lumos Tribrid mass spectrometer (Thermo Fisher Scientific, San José, CA, USA) coupled to a nanoACQUITY UPLC (Waters, Milford, MA, USA) as previously described (35, 36). Briefly, EV proteins were extracted with 100 µl PBS and 3% sodium deoxycholate (SDC): samples were lysed through five repeated freeze-thaw cycles (frozen in liquid nitrogen for 30 s and thawed at 50°C for 2 min), sonicated for 5 min (five cycles of 20 s with an interval between cycles of 40 s on ice) and samples were then clarified by centrifugation at 16,000 × *g* for 10 min at 4°C. Protein concentrations were determined by the bicinchoninic acid assay. After reduction with dithiothreitol and alkylation with iodoacetamide in 3% SDC, 10 µg of proteins extracted from extracellular vesicles were digested in 100 mM NH_4_HCO_3_ by sequencing grade modified trypsin (20:1, w/w, protein/enzyme) overnight (18 h) at 37°C. Digestion was halted by acidifying the reaction mixture (pH 2–3) with trifluoroacetic acid, and the digested samples were desalted and subjected to solid phase extraction with Waters HLB 1cc (30 mg) extraction cartridges. Peptides were eluted with 60% acetonitrile containing 0.3% of trifluoroacetic acid, and the eluted samples were dried and stored at −80°C until MS analysis. After the dried peptide digests were reconstituted in 0.1% formic acid, the peptide digests (equivalent to 0.4 μg of proteins) were loaded onto a C-18 trap column (0.1 × 40 mm) packed in house with Magic C-18 reverse-phase resin (5 µm; 100 Å; Michrom Bioresources) for 8 min at 2.5 μl/min in 99% solvent A (0.1% formic acid in water) and 1% solvent B (0.1% formic acid in acetonitrile). The peptides were then eluted from the trap column onto a C-18 analytical column (0.1 × 200 mm) packed in house with Magic C-18 reverse-phase resin (5 µm; 100 Å; Michrom Bioresources) and were separated at a flow rate of 0.3 μl/min using a multistep gradient as follows: 1% to 7% solvent B in 1 min; 7% to 25% solvent B in 75 min; and 25% to 35% solvent B in 15 min; 35% to 80% solvent B in 10 min. The column was subsequently washed for 5 min at 80% B and re-equilibrated at 99% A for 13 min. The column was kept at room temperature and the mass spectrometer was operated in data-dependent acquisition mode. The obtained MS/MS spectra were compared with the human UniProtKB database (HUMAN.20190801.fasta) using the Comet MS/MS search engine (version 2019.01 rev. 0) with fixed Cys alkylation and variable Met oxidations. Two missing cleavage sites in peptides were allowed for trypsin-restricted searches. Comet’s results were validated by PeptideProphet and ProteinProphet using an adjusted probability of >0.90 for peptides and >0.95 for proteins. For the identification of a protein, at least two peptides unique to the protein of interest had to be detected. Three independently obtained EV samples were analyzed. Proteins were considered exclusive or only present in EVHp+ when no peptide of that protein was found in any of the of EVHp- samples, and vice versa. Total peptide counts were used to compare the relative abundance of proteins. Proteins were considered enriched in EVHp+ when the ratio of mean peptide counts was greater than in EVHp-, and vice versa. Proteomics data were subjected to ontology and pathway analysis using the Analysis of Proteins through Evolutionary Relationships tool (PANTHER, http://www.pantherdb.org), and were classified based on biological processes and pathway categories.

### Scanning electron microscopy (SEM)

Samples were fixed using glutaraldehyde 2.5% in a proportion 1:1 with the sample. Each fixed EV sample (15 μl) was placed on a 200-mesh nickel grid (CF200-N1,Carbon Film Ni, Electron Microscopy Sciences, Hatfield, PA, USA) for 2 min, then the excess liquid was absorbed with filter paper. Subsequently, the grid was washed with water for 1 min. Then 15 μl of phosphotungstic acid (0.1%) were added and the mixture was incubated for 30 s before absorbing the excess liquid with filter paper. Finally, the grid was washed again with water for 2 min. The images were acquired with a High Resolution Scanning Electron Microscope, INSPECT‐F50 (Thermo Fisher Scientific, FEI, Holland), with a STEM detector.

### Statistical analysis

All data are expressed as mean ± standard error of mean of results from at least three independent experiments. The data from the characterization of EVHp- and EVHp+ were analyzed using the unpaired t-test. The rest of the data, in which three or more groups were compared, were analyzed using One-way ANOVA with multiple comparisons. Significance (p-value) was set at the nominal level of p < 0.05 or less. All data were processed using GraphPad Software (http://www.graphpad.com).

## Results

### *H. pylori* infection increases the secretion of extracellular vesicles from non-tumorigenic gastric epithelial GES-1 cells

EVs are implicated in many pathophysiological processes, such as pathogen-related diseases and cancer (24). Specifically, EVs can contribute to the distribution of molecules originating from the pathogen or host molecules associated with inflammation during an infectious disease. Regarding *H. pylori*, little is known about the changes that occur in EV-mediated cell-to-cell host communication during infection. Furthermore, it is not known whether *H. pylori* infection alters aspects of host cell-derived EVs, such as the number of released EVs and/or the content.

**Figure 1:**
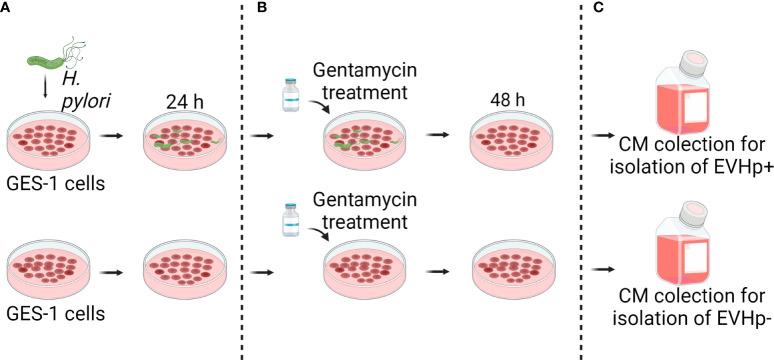
Experimental model to obtain conditioned medium from GES-1 infected or not with *H. pylori*. **(A)** GES-1 cells were infected or not with *H. pylori* for 24 h and then **(B)** treated with gentamycin. **(C)** 48 hours later, conditioned media from GES-1 infected or not with *H. pylori* (EVHp+ and EVHp-, respectively) were collected to isolate EVs by ultracentrifugation.

To evaluate the changes that occur in EVs released from gastric host cells during *H. pylori* infection, we isolated and characterized EVs from GES-1 cells infected or not with *H. pylori* (EVHp+ and EVHp-, respectively). Briefly, cells were infected or not with *H. pylori* for 24 h (MOI:100), and then treated with gentamycin to eliminate the bacteria. After an additional 48 h in culture, the conditioned medium was collected and used for EV isolation by ultracentrifugation (**Figure 1**). The purified EVs were analyzed to quantify the number of particles released, their size and the total protein content. In addition, exosome marker proteins and negative controls were identified by Western blotting of EVs and parental cell lysates.

**Figure 2:**
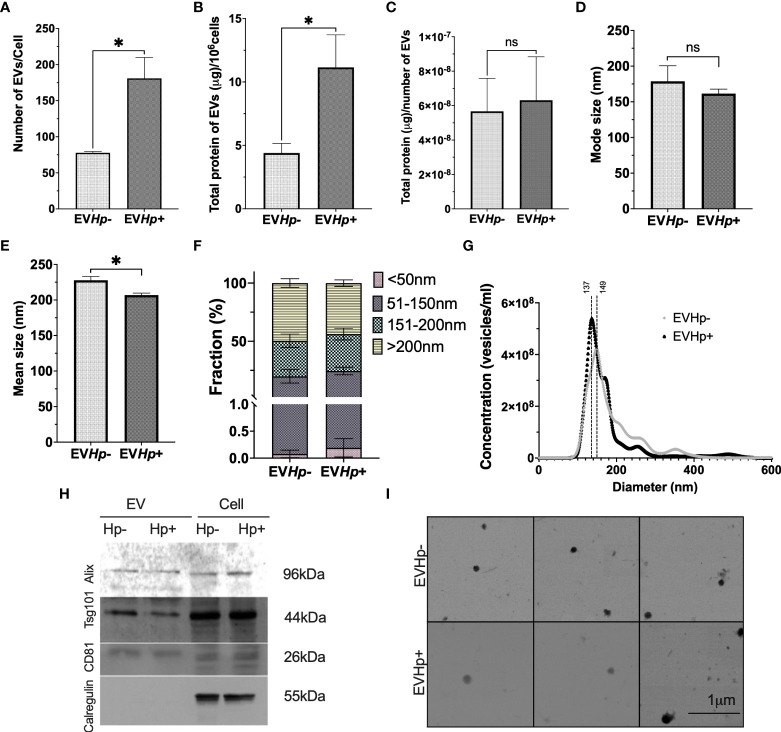
*H. pylori* promotes the release of EVs from GES-1 cells. EVs were isolated by ultracentrifugation from conditioned medium of cultured GES-1 cells infected or not with *H. pylori* (EV*Hp*+ and EV*Hp-*, respectively). The isolated EVs were analyzed by Nano Tracking Analysis (NTA) and Western blotting: **(A)** Number of EVs released per cell; **(B)** total protein content in EVs released per 10^6^ cells; **(C)** total protein content in EVs per particle; **(D)** mode size; **(E)** mean size; **(F)** percentage of vesicles belonging to the <50 nm, 50-150 nm, 150–200 nm and >200 nm fractions; **(G)** Vesicle concentration versus size (nm). Results shown were averaged from four independent experiments. Statistically significant changes (p value less than 0.05) is indicated as *, ns, not significant. **(H)** Western blots for the exosomal markers. Alix, Tsg101, CD81 and the ER control Calregulin of cell and EV lysates. An image representative of results from three experiments is shown. **(I)** Electron Microscopy images of EVHp- (top) and EV*Hp*+ (bottom) preparations were taken, to corroborate EV shape and size. The images revealed spherical structures of 50-150 nm in diameter for both types of EVs. EV, Extracellular vesicles; Cell, cell lysate. Hp-, no infection with *H. pylori*; Hp+, infection with *H. pylori*.

*H. pylori* infection increased the number of EVs released from GES-1 cells more than 2-fold, compared to cells without infection (**Figure 2A**), as determined by NTA. The mean number of EVs released per cell under basal conditions was ~ 80, whereas for the cells infected with *H. pylori* this value increased to ~ 180. The relevance of EVs depends amongst other things on their cargo protein content. Therefore, the total protein concentration in EVs was determined. We observed that the total protein content in EVs released per 10^6^ cells increased in EVHp+ compared to EVHp- (**Figure 2B**). To determine whether *H. pylori* infection was causing an increase in the protein content per EV or rather an increase in the total number of EVs released, we calculated the protein content per EV particle and observed no change (**Figure 2C**). Thus, *H. pylori* infection increases the number of EVs released by cells, but does not increase the relative concentration of the protein cargo per EV.

In addition, infection with *H. pylori* caused a slight decrease in the mode and mean size of EVs (**Figures 2D, E**). The latter is also reflected in **Figure 2F**, in which EVs were separated into size categories, revealing that in EVHp+, there is a higher percentage of vesicles smaller than 50 nm and between 50 nm and 150 nm. In addition, this also can be seen in **Figure 2G**, where different size distributions were observed. For EVHp- the highest peak is at 149 nm, whereas for EVHp+ the highest peak is at 137 nm. Also, a higher EV concentration was detected for EVHp+. To ensure that these results were not simply an artifact attributable to the purification by ultracentrifugation, EVs were also isolated from GES-1 cells infected or not with *H. pylori* using Exo-spin™ columns (Cell Guidance Systems; **Supplementary materials**). As for EVs isolated by ultracentrifugation, an increase in the number of released EVs and a slight, albeit not statistically significant, decrease in the mean size of EVs from GES-1 cells infected with *H. pylori* compared to EVs from uninfected cells (**Supplementary Figure 1**) was observed using Exo-Spin™ columns.

Moreover, according to the Exocarta database (37), Alix, Tsg101 and CD81 are included in the list of the 100 most frequently identified proteins in exosomes and are considered exosomal markers. The presence of Alix, Tsg101 and CD81 along with Calregulin, a control for endoplasmic reticulum contamination, were evaluated by western blotting. As expected, in EVHp- and EVHp+, Alix, Tsg101 and CD81 were detected, while Calregulin was absent (**Figure 2H**).

Finally, the size and shape of the EVs were analyzed by SEM (**Figure 2I**). EVs from both samples (EVHp- and EVHp+) were identified as spherical structures of diameters ranging from 50 to 150 nm, with mean values of 110 nm ±14 nm and 110 ±41 nm for EVHp- and EVHp+, respectively.

### EVs from *H. pylori*-infected GES-1 cells increase the levels of IL-23 in GES-1 cells

**Figure 3:**
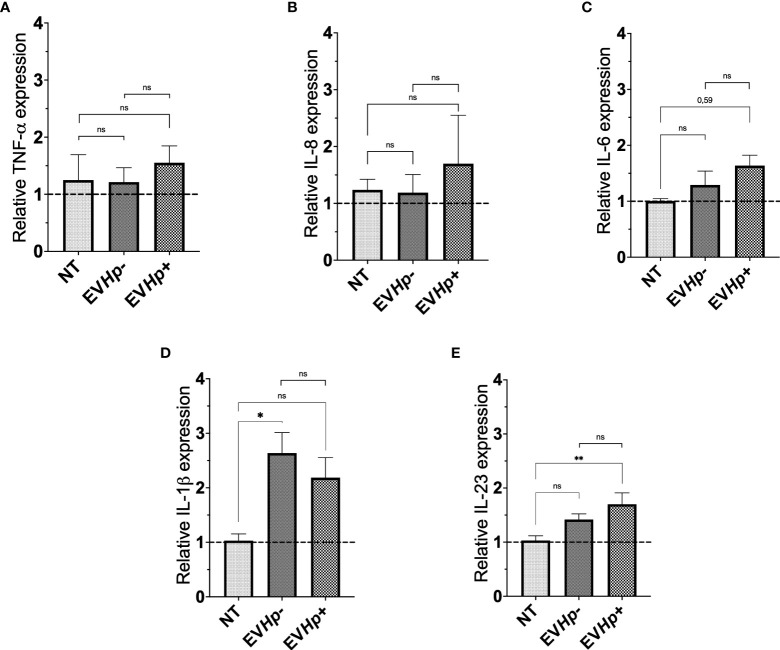
Expression of cytokines in non-tumorigenic gastric GES-1 cells without stimulation (NT: no-treatment), and stimulated with EVHp- or EVHp+ for 24 h. Relative expression levels of **(A)** TNF-α; **(B)** IL-8; **(C)** IL-6; **(D)** IL-1β and **(E)** IL-23 measured by RT-qPCR averaged from four independent experiments are shown. The hatched horizontal line indicates a relative gene expression =1 (no change). Values above that level indicate an increase in the relative expression. Statistically significant changes (p value less than 0.05 or 0.01) are indicated as * or **, respectively; ns, not significant.

Infection with *H. pylori* increases the levels of the pro-inflammatory cytokines (12). In order to show that EVs from GES-1 infected with *H. pylori* cells participate in the spread of infectious disease, the mRNA levels of proinflammatory cytokines TNF-α, IL-8, IL-6, IL-1β and IL-23, in GES-1 (**Figure 3**) and AGS cells (**Supplementary Figure 2**) stimulated for 24 h with EVHp- or EVHp+ were measured by RT-qPCR. The treatment of GES-1 cells with EVHp- or EVHp+ did not induce changes in the levels of TNF-α and IL-8 (**Figures 3A, B**). IL-6 mRNA cytokine levels showed a tendency to increase with EVHp+ stimulation only (**Figure 3C**). IL-1β mRNA cytokine levels increased with EVHp- stimulation, but not significantly with EVHp+ (**Figure 3D**). On the other hand, IL-23 mRNA levels increased significantly only following EVHp+ stimulation (**Figure 3E**). This increase at the mRNA level was corroborated by measuring IL-23 also at the protein level in the supernatants of GES-1 cells stimulated with EVHp+ (**Supplementary Figure 3**). This analysis revealed a more than a two-fold increase compared to the control condition in conditioned media isolated 24 h post-treatment. Alternatively, in the AGS gastric cancer cells, the incubation with EVHp- or EVHp+ did not induce significant increases in the mRNA levels of any of the pro-inflammatory cytokines measured (**Supplementary Figure 2**). Although the infection with *H. pylori* increases the release of EVs, the cells were stimulated *in vitro* with equal amounts of EVHp- or EVHp+. Therefore, the minor increases in the mRNA levels of pro-inflammatory cytokines observed after stimulation with EVHp+ as compared with EVHp- (in particular IL-23) would likely be enhanced by increasing the EVHp+ dose.

### EVs from *H. pylori*-infected GES-1 cells decrease the viability of non-tumorigenic gastric GES-1 cells

**Figure 4:**
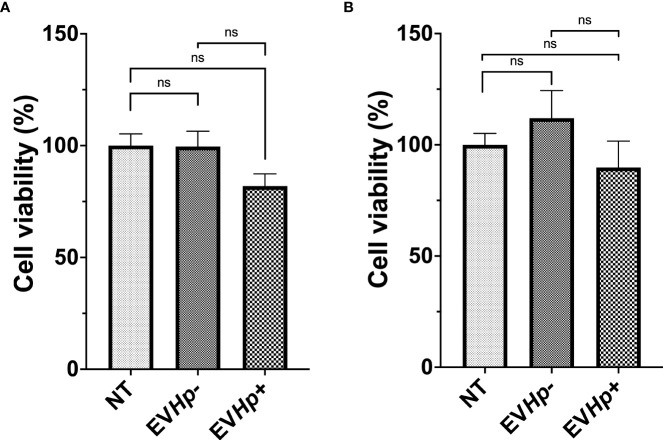
Viability of non-tumorigenic gastric GES-1 and gastric cancer AGS cells without stimulation (NT: no-treatment) and after incubation with EVHp- or EVHp+ for 24 h. **(A)** GES-1 cells and **(B)** AGS cells were incubated with EV*Hp*- or EV*Hp*+ for 24 h. Then, cell viability was determined by Trypan Blue staining. ns, not significant.

Gastric mucosa lesions observed in patients infected with *H. pylori* have been associated with the reduction in cell viability and cell death induced by *H. pylori in vitro* (38, 39). Furthermore, in the gastric cell population that survives after *H. pylori* infection, adaptive responses are triggered that favor cell survival and increase cell proliferation, characteristics that are associated with malignancy (17). Since EVs can function as propagators of damage during diseases caused by pathogens, we evaluated GES-1 and AGS cell viability after 24 h of stimulation with EVHp- or EVHp+. GES-1 cell viability was not affected by stimulation with EVHp-. For GES-1 cells stimulated with EVHp+, cell viability tended to decrease; however, this difference was not statistically significant (**Figure 4A**). Alternatively, the incubation of tumorigenic AGS cells with 12 μg EVHp- or EVHp+ did not affect viability (**Figure 4B**).

### Cell migration and invasion of epithelial gastric cells increases after incubation with EVs from *H. pylori*-infected GES-1 cells

**Figure 5:**
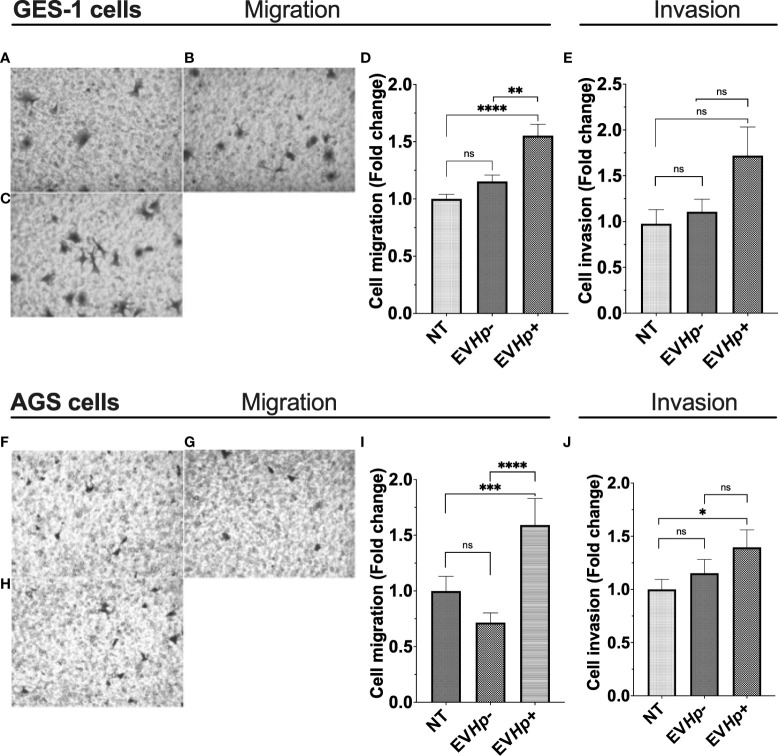
EVs isolated from GES-1 infected with *H. pylori* increase cell migration and invasion of non-tumorigenic gastric GES-1 and gastric cancer AGS cells. GES-1 **(A–E)** and AGS **(F–J)** cells were either left without stimulation (NT: no-treatment) or stimulated with EVHp- or EVHp+ for 24 h. Then, cell migration and invasion were determined in Transwell and Matrigel assays, respectively. Representative images are shown for GES-1 cells that migrated without stimulation **(A)**, following stimulation with EVHp- **(B)** or EVHp+ **(C)**; **(D)** quantification of GES-1 cell migration averaged from four independent experiments; **(E)** quantification of GES-1 cell invasion averaged from four independent experiments. Representative images are shown for AGS cells that migrated without stimulation **(F)**, following stimulation with EVHp- **(G)** or EVHp+ **(H)**; **(I)** quantification of AGS cell migration from three independent experiments; **(J)** quantification of AGS cell invasion averaged from three independent experiments. Statistically significant differences (p values less than 0.05, 0.01, 0.001 and 0.0001) are indicated as *, **, *** or ****, respectively; ns, not significant.

Cell migration and invasion are important characteristics of malignancy in the context of cancer. Studies indicate that EVs from highly metastatic cancer cells are able to increase migration and invasion of less metastatic cancer cells (40, 41). Furthermore, exosomes from fibrosarcoma cells increase cell migration in an autocrine manner (42). On the other hand, *H. pylori* infection increases the migration of gastric epithelial cells (39, 43). Therefore, cell migration and invasion of the non-tumorigenic GES-1 and tumorigenic AGS and Hs746T cells were determined 24 h after incubation with EVHp- or EVHp*+.* The incubation of GES-1, AGS and Hs746T cells with EVHp- neither increased their cell migration compared to cells without treatment (**Figures 5A, B, D, F, G, I**) or their ability to invade (**Figures 5E, J** and **Supplementary Figure 4**). Alternatively, EVHp*+* induced a substantial increase (>50%) in the migration of GES-1 (**Figures 5C, D**) and AGS cells (**Figures 5H, I**), in comparison to controls, while the increase was only modest (not statistically significant) for Hs746T cells (**Supplementary Figure 4**). However, in the latter case, the increase was significant in comparison HsT467 cells treated with EVHp-. Moreover, upon incubation of GES-1, AGS and Hs746T cells with EVHp+, the invasion of AGS cells increased while only a tendency towards increased invasion was observed for GES-1 and Hs746 cells (**Figures 5E, J** and **Supplementary Figure 4**). Again, in the latter case, the increase was significant in comparison to Hs746 cells treated with EVHp-.

### EVs from *H. pylori*-infected GES-1 cells increase transendothelial cell migration of epithelial gastric cells

**Figure 6:**
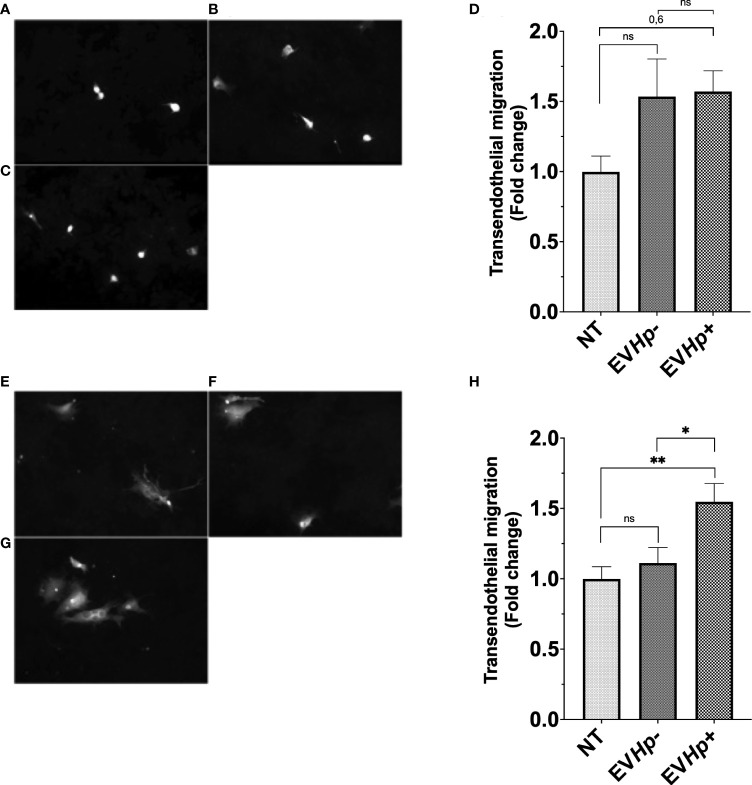
EVs isolated from GES-1 cells infected with *H. pylori* increase transendothelial migration of non-tumorigenic gastric GES-1 and gastric cancer AGS cells. GES-1 **(A–D)** and AGS **(E–H)** cells were either left without stimulation (NT: no-treatment) or stimulated with EVHp- or EVHp+ for 24 h. Then, cell migration across an impermeable endothelial monolayer was determined. Representative images are shown for GES-1 cells that transmigrated without stimulation **(A)**, following stimulation with EVHp- **(B)** or EVHp+ **(C)**; **(D)** quantification of GES-1 endothelial transmigration averaged from three independent experiments. Representative images are shown for AGS cells that transmigrated without stimulation **(E)**, following stimulation with EVHp- **(F)** or EVHp+ **(G)**; **(H)** quantification of AGS endothelial transmigration averaged from three independent experiments. Statistically significant differences (p value less than 0.05 and 0.01) are indicated as * or **, respectively; ns, not significant.

To obtain information about whether the EVs released from GES-1 gastric epithelial cells infected with *H. pylori* may be important in processes, such as intravasation and/or extravasation, both processes related to the cascade of events that precede metastasis, we performed *in vitro* transendothelial migration assays. For this, GES-1 and AGS cells stimulated for 24 h with EVHp- or EVHp+ were seeded on top of an impermeable monolayer of EA.hy926 endothelial cells previously seeded in transwells. The cells to be evaluated were stained with CellTracker ™. Therefore, those that managed to cross the endothelial cell monolayer could be tracked on the other side of the transwell thanks to their fluorescence. The incubation of GES-1 (**Figures 6A, B, D**) and AGS cells (**Figures 6E, F, H**) with EVHp- did not increase the transendothelial cell migration. Alternatively, upon incubation with EVHp+, a tendency toward increased transendothelial migration was detectable for GES-1 cells (**Figures 6C, D**), while for AGS cells (**Figures 6G, H**) the increase was significant.

### The formation of vascular networks increases after incubation with EVs isolated from GES-1 cells infected with *H. pylori*

**Figure 7:**
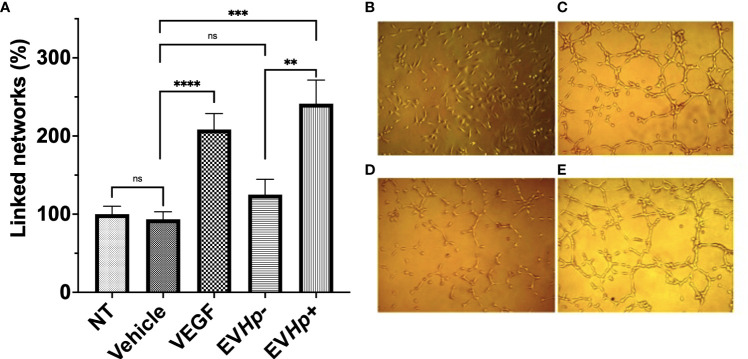
EVs released from *H. pylori* infected GES-1 cells increase linked networks formed by endothelial EA.hy926 cells. The formation of linked networks by EA.hy926 cells was determined under the following conditions: NT, Vehicle, 50 ng/mL VEGF as a positive control, 10 µg EVHp- or 10 µg EVHp+. Values shown were averaged from four independent experiments **(A)**. Representative images of the results obtained under the conditions indicated are shown: **(B)** NT; **(C)** VEGF; **(D)** EVHp- and **(E)** EVHp+. Statistically significant differences (p value less than 0,01, 0.001 and 0.0001) are indicated as **, *** or ****, respectively; ns, not significant.

Cell to cell communication through EVs has been shown to be important in angiogenesis, one of the hallmarks of cancer. Specifically, the stimulation of endothelial cells with exosomes reportedly increases angiogenesis (44). On the other hand, *H. pylori* infection has been previously described to affect endothelial cells and the vascular system (45). Therefore, to determine whether EVs released from GES-1 cells infected with *H. pylori* could modulate processes, such as vasculogenesis, the formation of vascular networks by endothelial EA.hy926 cells was evaluated *in vitro*. The stimulation with EVHp- did not cause significant changes in the percentage of linked networks, while the exposure to EVHp+ increased more than 2-fold the number of such networks formed by EA.hy926 cells, as compared to the vehicle (**Figure 7**). Of note, the observed induction was even greater that that observed with the positive control VEGF.

### EVs released by *H. pylori*-infected gastric GES-1 do not include CagA or any other recognizable *H. pylori* epitopes

**Figure 8:**
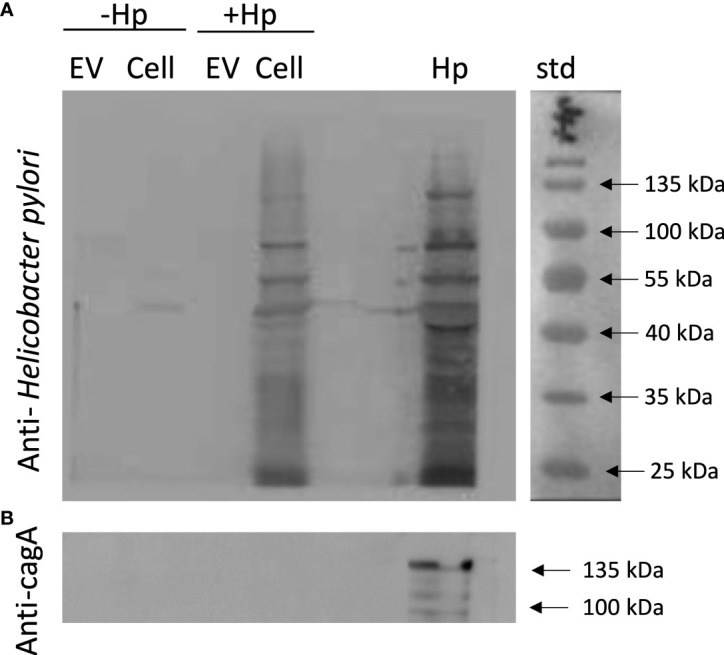
EVs released from *H. pylori*-infected gastric GES-1 cells neither include CagA or any other recognizable *H. pylori* epitopes. EVs and lysates of GES-1 cells, infected or not-infected cells with *H. pylori*, were evaluated by Western blotting using anti-Hp **(A)** and anti-CagA antibodies **(B)**. EV, Extracellular vesicles; Cell, cell lysate; Hp, *H. pylori* lysate; std, molecular-weight standard; Hp-, no infection with *H. pylori*; Hp+, infection with *H. pylori*.

There is evidence indicating that EVs during infection with a pathogen serve as vehicles for transporting pathogen-specific molecules or host cell-derived molecules in the context of infection (46). To rule out that in our experimental model, the EVs from GES-1 cells infected with *H. pylori* contain proteins derived from the pathogen, lysates of EVs and cells infected or not with *H. pylori*, were evaluated by Western blotting using an anti-CagA antibody and a polyclonal anti-*H. pylori* antibody. This experiment revealed that neither bands corresponding to CagA, or to any *H. pylori* epitope, were detectable in EV samples (**Figure 8**). Therefore, the biological changes in recipient cells induced by the incubation with EVHp+ are most likely attributable to alterations in the presence of host cell-derived molecules, as a consequence of *H. pylori* infection.

### Proteomics analysis by mass spectrometry of EVHp- and EVHp+ reveals differences in the protein composition of vesicles

**Figure 9:**
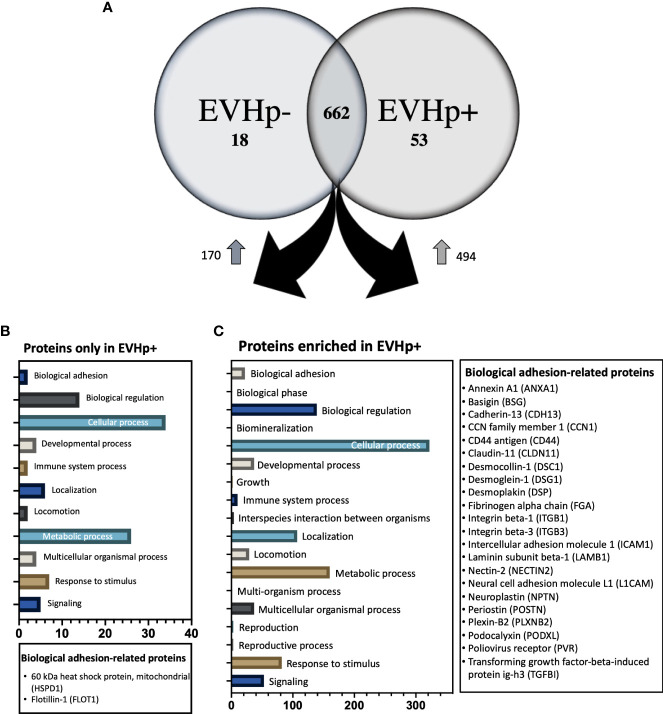
Proteomics analysis by mass spectrometry of EVHp- and EVHp+. **(A)** 662 proteins were detected as common to EVHp- and EVHp+, while 18 proteins were found exclusively in EVHp- and 53 proteins exclusively in EVHp+. Relative abundance analysis revealed 170 proteins enriched in EVHp- compared to EVHp+, whereas 494 proteins were enriched in EVHp+ compared to EVHp-. Gene ontology (GO) categories to which proteins were assigned as ‘Biological Processes’ in proteins **(B)** exclusively detected in EVHp+ and **(C)** enriched in EVHp+. Proteins associated with ‘biological adhesion’ are individually listed for each analysis.

When gastric epithelial and endothelial cells were incubated with equal concentrations of EVHp- and EVHp+, the observed effects were generally greater with EVHp+. Moreover, our Western blotting data suggest that these differences are not due to the transfer of pathogen-derived proteins by EVHp+. Therefore, the differential biological effects observed following treatment of cells with EVHp+ were potentially due to alterations in the content of host-derived proteins in EVs following infection with *H. pylori*. Since proteins are one of the most important components involved in EV-mediated cell-cell communication, those present in EVHp- and EVHp+ were compared by mass spectrometry analysis. The results identified 662 proteins present in both samples (**Figure 9A**), while 53 proteins were exclusively detected in EVHp+ (**Figure 9A** and **Supplementary Table 2**) and 18 proteins exclusively in EVHp- (**Figure 9A** and **Supplementary Table 3**) samples.

A relative abundance analysis was performed for the 662 proteins found in both samples. According to this, 494 proteins were enriched in EVHp+ compared to EVHp- (**Figure 9A** and **Supplementary Table 4**), whereas 170 proteins were enriched in EVHp- compared to EVHp+ (**Figure 9A**). Of the proteins enriched in EVHp+, differences were significant for 4 (T-test) (data not shown): hornerin (HRNR), heat shock 70 kDa protein 1A (HSPA1A), intercellular adhesion molecule 1 (ICAM-1) and 5’-3’ exonuclease PLD3 (PLD3). To further analyze the possible functions in which the proteins detected exclusively or enriched in EVHp+ may be involved, these were analyzed by Gene ontology (GO) enrichment analysis using the PANTHER classification system (**Figures 9B, C**) and the GeneCards database (47). The GO analysis showed that 2 components of the biological process ‘cell adhesion’ were unique to EVHp+ and were not present in EVHp-, namely 60 kDa heat shock protein, mitochondrial (HSPD1) and Flotillin-1 (FLOT1) (**Figure 9B**). FLOT1 is a protein that plays a role in vesicle trafficking and cell morphology, as well as a scaffolding protein, important in transmembrane signaling and cell adhesion (48), while HSPD1 functions as a molecular mediator of alpha 3 beta 1 integrin activation (49). In addition, HSPD1 mRNA and protein levels were higher in GC tissues compared to the control, and knockdown of HSPD1 inhibited cell mobility (50).

Among the exclusive proteins in EVHp+, those found with a greater number of peptide counts were Sequestosome-1 (SQSTM1), Amyloid-beta precursor protein (APP) and Tyrosine-protein kinase JAK1. SQSTM1 binds ubiquitin and regulates activation of the nuclear factor kappa-B (NF-kB) signaling pathway (51). APP is a cell surface receptor and transmembrane precursor protein, initially associated with Alzheimer disease; however, recent reports indicate that it harbors antimicrobial activity (52). Finally, JAK1 when activated phosphorylates STAT proteins, important for the expression of genes that mediate inflammation, epithelial remodeling, cancer progression and metastasis (53, 54). Other proteins important in biological processes, found exclusively in EVHp+, were IL-6 receptor subunit beta (IL6ST), Mast/stem cell growth factor receptor Kit (KIT) and TNF receptor superfamily member 10B (TNFRSF10B). The IL6ST is a signal transducer shared by many cytokines, including IL-6. Binding of IL-6 the IL-6R induces activation of the JAK-MAPK and JAK-STAT3 signaling pathways (55). KIT is a receptor tyrosine kinase that, after activation, phosphorylates multiple intracellular proteins relevant to cell proliferation, differentiation, migration and apoptosis (56). TNFRSF10B is a member of the TNF-receptor superfamily and contains an intracellular death domain. This receptor can be activated by tumor necrosis factor-related apoptosis inducing ligands (TNFSF10/TRAIL/APO-2L) and triggers apoptosis in recipient cells (57).

Moreover, 22 proteins of the biological process ‘cell adhesion’ were enriched in EVHp+ compared to EVHp-, including Integrin β1 (ITGB1) and Integrin β3 (ITGB3) (**Figure 9C**). On the other hand, some of the proteins enriched in EVHp+ (**Supplementary Table 4**) are important for the biogenesis and release of exosomes (CD81, Rab5, CD63, Annexin A2, Annexin A5, HSPA1, HSP90, Rab-7a and Rap-1b) (37), cell adhesion and migration (Integrin β1, ICAM-1 and Syntenin-1) (58), as well as cell proliferation and metastasis (RhoA transforming protein, RHOA) (59). The presence and enrichment of some of these proteins in EVHp+ in comparison to EVHp- was confirmed by Western blot analysis (**Supplementary Figure 5**). Although the enrichment was generally not very high (only 2-fold in some cases), we also observed a greater than two-fold increase in EV release from *H. pylori*-infected cells (**Figure 2A**). In combination, this can be expected to increase the presence of these EV-associated proteins and their effects in recipient cells.

In some cases, such as for Integrin β1 and RhoA, the band detected in EVs had a higher molecular size than observed in the extracts. This increase in size is consistent with modification by monoubiquitylation or monosumoylation, processes known to be involved in protein sorting into extracellular vesicles (60, 61).

Finally, our laboratory has recently shown that infection of gastric cells with *H. pylori* increases the expression of hypoxia induced factor-1α (HIF-1α) (28), a transcription factor considered crucial for inducing metabolic changes associated with the development of cancer. Interestingly, among the proteins whose expression is regulated by HIF-1α (62), we detected by mass spectrometry analysis some enriched in EVHp+, such as RhoA, Alpha-enolase (ENO1), Glyceraldehyde-3-phosphate dehydrogenase (GAPDH), Insulin-like growth factor II (IGF2); L-lactate dehydrogenase (LDHA) chain A, PKM pyruvate kinase (PKM) and Vimentin.

## Discussion

EVs are an important mechanism of cell-to-cell communication, relevant in inflammation, pathogen-related diseases and cancer (44, 63). In pathogen-caused diseases, EVs participate in the spread of the infection, as has been reported for those caused by *Mycobacterium tuberculosis, Salmonella, Chlamydia pneumoniae*, among others (26). This effect of EVs during infection is attributed to their ability to contribute to the distribution of molecules originating either directly from the pathogen itself or host-derived molecules associated with inflammation (25, 46). Furthermore, EVs promote several hallmarks of cancer beyond inflammation, including cell proliferation, angiogenesis and cell migration (44, 63). On the other hand, infection with *H. pylori* is considered the main risk factor for the development of gastric cancer. Therefore, EVs released after infection with *H. pylori* are likely to increase the risk of developing GC in patients infected with this pathogen. However, to date little is known in this respect.

Here, the effect of *H. pylori* infection on the EVs released from non-tumorigenic gastric GES-1 cells was evaluated. Moreover, the effects of EVs isolated from GES-1 infected with *H. pylori* on several cellular traits relating to malignancy in the recipient cells were determined. After infection with *H. pylori*, GES-1 cells released 50% more EVs of slightly reduced size to the extracellular medium (**Figure 2**). This interestingly agrees with the fact that in inflammatory diseases, such as Sjögren’s syndrome, systemic lupus erythematosus and rheumatoid arthritis, the circulating EV levels in patients are significantly higher than those in healthy controls (25, 64, 65). In addition, GC patients have a significantly greater number of total EVs circulating in the bloodstream (66). The smaller mean EV size (for EVHp- and EVHp+) determined by SEM than the NTA data, is likely attributable to the dehydration of the vesicles that occurs under conditions used to fix samples for analysis, as has been reported elsewhere (67).

Infection with *H. pylori* is known to increase the levels of pro-inflammatory cytokines (12). However, EVs from GES-1 cells infected with *H. pylori* seem to have little effect on the levels of pro-inflammatory cytokines in recipient cells (see **Figure 3**). A small, but significant, increase in the mRNA levels was only detected for IL-23 in GES-1 cells after incubation with EVHp+, and this was corroborated by measuring protein levels of the same cytokine in ELISA assays (**Supplementary Figure 3**). However, although only a modest increase, continuously elevated levels of pro-inflammatory cytokines, as observed in chronic inflammation, have been identified as one of the causes of gastric carcinogenesis associated with *H. pylori* infection (12). Interestingly, IL-23 has been shown to be important in the context of *H. pylori* infection and in the progression of gastric cancer (13, 14). Moreover, IL-23 was recently found to increase cell migration and invasion of gastric cancer cells by inducing epithelial-to-mesenchymal transition *via* the STAT3 pathway (68). It is intriguing to speculate that this may represent a possible mechanism to explain how EVHp+ increase cell migration and invasion, as reported on here in our studies. However, it is also clear from our studies that this cannot be the only mechanism to explain the changes in behavior of cells incubated with EVHp+. Although unexpected, the increase in the pro-inflammatory cytokine IL-1 β mRNA levels observed with EVHp- stimuli at the concentration used may relate to the role of EVs in autocrine/paracrine cell to cell communication. In particular, the different responses observed for cytokine levels upon stimulation with EVHp+ or EVHp- is likely attributable to the differential protein composition observed for EVHp+ compared to EVHp- (**Figure 9**).

Viability of the non-tumorigenic GES-1 cells decreased after stimulation with EVHp+, although this difference was not statistically different (**Figure 4**). This resembles previous observations from our laboratory (69) and by Wang et al. (38), in which induction of apoptosis was observed in GES-1 cells after 24 h of infection with *H. pylori*. Moreover, it has been reported that inflammation due to *H. pylori* infection increases the release of free radicals and damages the DNA of epithelial cells, which also triggers apoptosis (70). This result is relevant, since it has been suggested that in the population of cells that survive an initial *H. pylori* infection, adaptive responses are triggered that favor cell survival and increase cell proliferation, characteristics important in the development of cancer (17). For cells that remain viable after incubation with EVHp+, a similar outcome might be expected.

The migration of GES-1 cells increased significantly following stimulation only with EVHp+. In addition, we found that only incubation with EVHp+, but not EVHp-, increased migration of AGS gastric cancer cells (**Figure 5**). Furthermore, incubation of non-tumorigenic gastric GES-1 and gastric cancer AGS cells with EVHp+ increased cell invasion and transendothelial migration (**Figures 5**, **6**). In a second gastric cancer cell line, Hs746T, the incubation with EVHp+ induced a tendency towards increased levels of cell migration and invasion when compared with non-treated cells, but a substantial, significant increase in comparison to EVHp- treated cells (**Supplementary Figure 4**). Although it may seem unexpected that GES-1 cells, despite being non-cancer cells, have the ability to migrate and invade in basal conditions, it has been described that cell migration occurs and is an important process in gastric epithelial cells, since it aids in the regeneration of the gastric epithelium after damage (71). Regarding the invasiveness of GES-1 cells, it remains unknown whether this is also important for the function of gastric epithelial cells. However, GES-1 cells are reported to invade as assessed by *in vitro* assays (72–74). Increased cell migration and invasion of gastric cells, both cancer-related characteristics, after stimulation with EVHp+, may favor the progression of this pathology.

Cell-to-cell communication through EVs has also been shown to increase angiogenesis (44). In addition, *H. pylori* infection has been reported to affect endothelial cells and the vascular system (45). We observed that incubation of EA.hy926 endothelial cells with EVHp+ increased linked network formation (**Figure 7**). The causal relationship between infection by *H. pylori* and angiogenesis is not entirely clear, although inflammation appears to be relevant in this context. Chang et al. (2005) reported that *H. pylori*-induced COX-2 expression enhances angiogenesis *via* TLR2 and TLR9 (75). Furthermore, Liu et al. (2016) proposed that *H. pylori*-induces VEGF expression mediated by COX-2 *via* activation of the Wnt/beta-catenin pathway (76). However, we did not find these specific molecules in our mass spectrometry analysis. Thus, other EV molecules may be involved in these events. On the other hand, Xia et al. (2020), reported that the incubation of HUVEC endothelial cells with exosomes from GES-1 cells infected with *H. pylori* induced a decrease in the proliferation, migration and formation of linked networks by these cells (77). Although the results regarding the formation of linked networks in endothelial cells are opposite to those found in this study, it should be noted that the protocol used to evaluate the effects of EVs from cells infected with *H. pylori* on endothelial cells reported by Xia et al. (77) was different from the one used here. The endothelial cells used in that study were HUVECs, while here we employed EA.hy926 cells; the infection with *H. pylori* in their study was for 2 h, while in our case we infected cells for 24 h; after the infection with *H. pylori*, cells were not treated with antibiotics to eliminate the bacteria, as was the case in our study. The latter step is crucial to exclude the possible co-purification of EVs from GES-1 cells with Outer membrane vesicles (OMVs) from *H. pylori*. Therefore, it cannot be ruled out that the results obtained in the aforementioned previous study were mediated in part by the participation of *H. pylori* OMVs. In any case, the evidence presented here suggests that EVs generated in the context of infection with *H. pylori* are relevant in processes, such as angiogenesis/vasculogenesis.

Regarding the molecular mediators of the biological effects observed after incubating recipient cells with EVs from GES-1 cells infected with *H. pylori*, Shimoda et al. (2016) reported on the presence of *H. pylori* virulence factor CagA in extracellular vesicles isolated from the serum of *H. pylori*-positive patients (29). In our experiments, the biological effects observed in recipient cells after incubation with EVHp+ were not caused by CagA or other *H. pylori*-derived proteins, as demonstrated by Western blotting (**Figure 8**). Therefore, *H. pylori* infection causes changes in the composition of host-derived molecules in EVs, which in turn are responsible for the biological effects observed in recipient cells.

To better understand these changes, the protein composition of the EVs from GES-1 cells infected or not with *H. pylori* was determined by mass spectrometry (**Figure 9**). The results identified several proteins that are uniquely present or enriched in EVHp*+* compared to EVHp*-*, important for proinflammatory signaling (SQSTM1 and JAK1), cell adhesion and migration (ICAM-1, Integrin β1 and Syntenin-1), exosome biogenesis and release (CD81, Rab-5), as well as cell proliferation and metastasis (RhoA transforming protein). The enrichment of these proteins in EVHp+ in comparison with EVHp- was verified by Western blot analysis (**Supplementary Figure 5**). Somewhat unexpectedly, differences detected by this method were generally at most 2-fold if at all. None-the-less, bearing in mind that *H.pylori*-infected cells produce significantly greater numbers of vesicles, the consequences of these minor differences in protein composition will likely be amplified.

On the other hand, it has been described that infection of gastric cells with *H. pylori* increases the levels of HIF-1α (28). This transcription factor regulates the expression of different proteins related to angiogenesis, glucose metabolism and cell proliferation/survival (78–80). Among the proteins whose expression is regulated by HIF-1α, we detected some enriched in EVHp+, such as RhoA, ENO1, GAPDH, IGF2, LDHA, PKM and Vimentin. Some of these proteins, specifically RhoA, Vimentin, LDHA and are very important proteins in the development and progression of cancer (79). In particular, a high percentage of mutations in RhoA, which due to their location are believed to increase activity, were found in genetically stable gastric tumors (59). Thus, the likely increase in the expression of these proteins mediated by HIF-1α stabilization following *H. pylori* infection, could favor the expression and then inclusion of these proteins in the EVHp+. Additionally, in hypoxia, cells release EVs that induce therapy resistance in recipient cells, mediated by proteins such as PKM2 and HSP70 (81). Combined with the evidence that *H. pylori* infection increases HIF-1α levels in gastric cells, it is possible that EVs from *H. pylori*-infected cells could promote such a therapy resistance phenotype in recipient cells as well. This represents an intriguing possibility that merits further research in the future to explain how EVHp+ promotes in recipient cells the acquisition of characteristics associated with the development of gastric cancer. It is important to emphasize again that although the infection with *H. pylori* increases the release of EVs, in all the experiments the recipient cells were incubated with equal concentrations of EVHp- and EVHp+. These results suggest that in the context of an active infection with *H. pylori*, the greater release of EVs could exacerbate the biological effects observed here.

**Figure 10:**
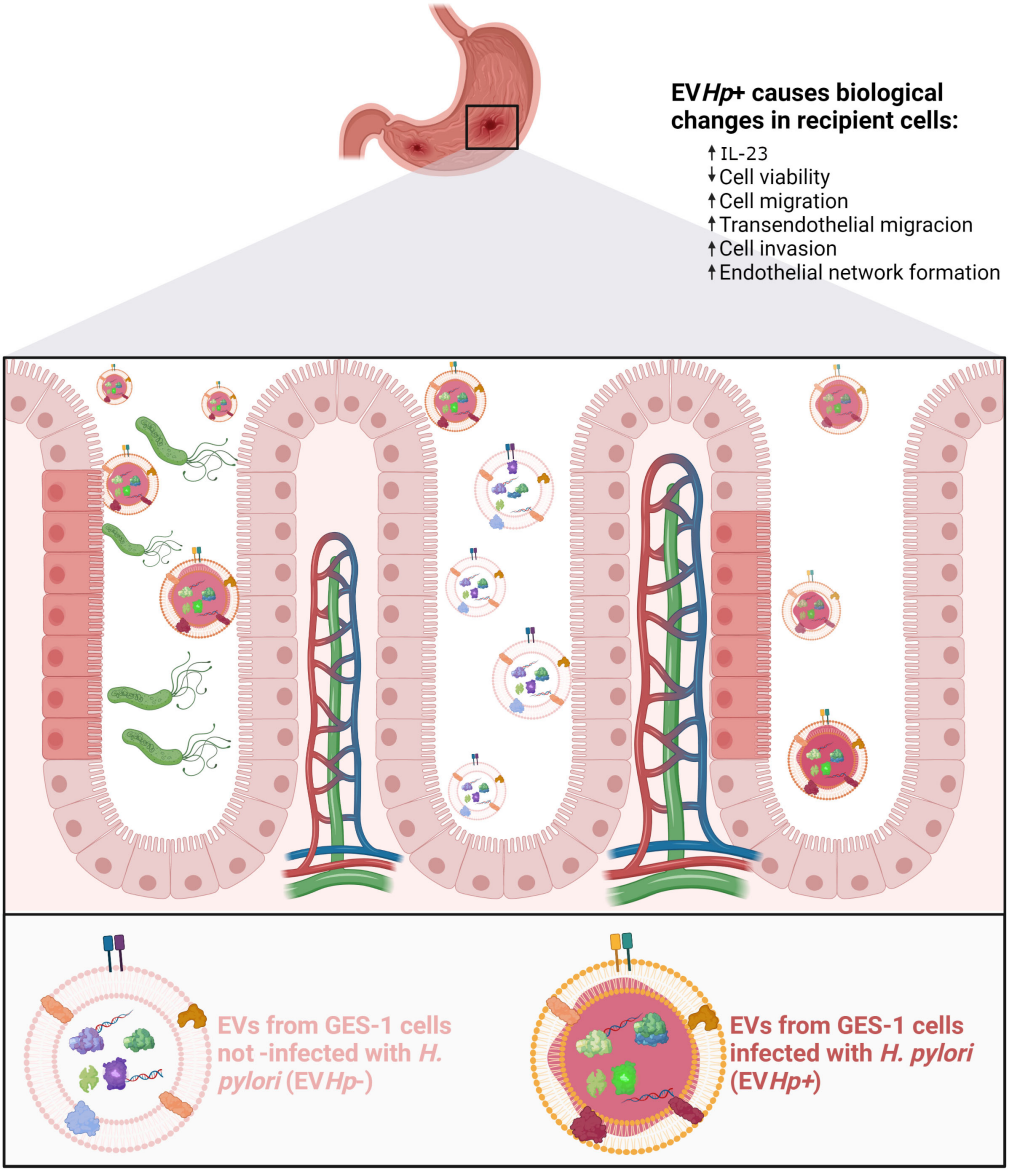
Schematic illustrating changes in recipient cells caused by EVs released from GES-1 cells infected with H. pylori. EVs released from GES-1 cells infected with *H. pylori* are different from EVs released by GES-1 cells under basal conditions. These differences are depicted as allowing them to induce effects associated with malignancy in recipient cells, such as decreased cell viability, increased levels of IL-23, increased migration/transendothelial migration/invasion. In addition, exposure of endothelial cells to such EVs is depicted as leading to changes in the vasculature (vasculogenesis). Created with http://BioRender.com.

In conclusion, intercellular communication between gastric cells mediated by EVs is altered after infection with *H. pylori* (see **Figure 10**), in a manner suggesting that such EVs provide an additional indirect mechanism by which *H. pylori* can trigger changes in the gastric epithelium and blood vessel endothelium that favor the development and progression of gastric cancer.

One of the limitations of this study is that we did not evaluate the role of extracellular vesicles liberated by gastric epithelial cells infected with *H. pylori in vivo* or using primary gastric cells. We performed all of our experiments using cell lines *in vitro*. This allowed us to evaluate the role of EVs from epithelial gastric cells infected with *H. pylori* in the communication with other gastric epithelial cells, gastric cancer cells and endothelial cells. Using this approach, our studies strongly suggest that these EVs produced by cells infected with *H. pylori* are able to change the behavior of a number of different types of cells present in the gastric environment in a manner that would favor the development and progression of gastric cancer. With this in mind, we are currently in the process of establishing *in vivo* models to study this phenomenon and confirm our results *in vivo*.

## Data availability statement

The original contributions presented in the study are included in the article/supplementary material. Further inquiries can be directed to the corresponding author.

## Author contributions

MG and AQ designed the study; MG, RB-R, and BS performed experiments; MV-V, JH, AC, and AQ supervised the study; MG and AQ wrote the manuscript; MG, RB-R, BS, JH, MV-V, AC, and AQ reviewed the manuscript. All authors contributed to the article and approved the submitted version.

## Funding

MG (ANID – National Ph.D. scholarship N° 21170292); RB-R (ANID – National Ph.D. scholarship N° 21200147); MV-V (UCEN CIP2019015); JH (NIH/NHLBI grants R01HL149685, P01 HL128203), AC (Fondecyt 1191928, FONDAP 15130011); AQ (Fondecyt 1210644, FONDAP 15130011).

## Acknowledgments

This work was possible thanks to the use of the Nanosight NS300 equipment (FONDEQUIP EQM160157) and the High Resolution Scanning Electron Microscope (Fondequip EQM170111). We also thank Cecilia Zuñiga and Herve Camus of the CEMC facility for their technical support relating to use of the ultracentrifuge (Sorvall WX+100) and the fluorescence microscope (Spinning disk Olympus IX81). We thank Dr. Karen Bolaños and Dr. Ana Riveros for their help in handling the samples for electron microscopy. The authors also thank Alejandra Sandoval for the use of her paid account to make the figures created with BioRender.com accessed on April 2022.

## Conflict of interest

The authors declare that the research was conducted in the absence of any commercial or financial relationships that could be construed as a potential conflict of interest.

## Publisher’s note

All claims expressed in this article are solely those of the authors and do not necessarily represent those of their affiliated organizations, or those of the publisher, the editors and the reviewers. Any product that may be evaluated in this article, or claim that may be made by its manufacturer, is not guaranteed or endorsed by the publisher.
