# Supplementary material for: Extracellular vesicles from gastric epithelial GES-1 cells infected with Helicobacter pylori promote changes in recipient cells associated with malignancy
Source: Front Oncol. 2022 Oct 12;12:962920. doi: 10.3389/fonc.2022.962920 (PMC9596800; doi:10.3389/fonc.2022.962920)
Supplement: Supplementary file 1 [file DataSheet_1.docx]

Supplementary Material


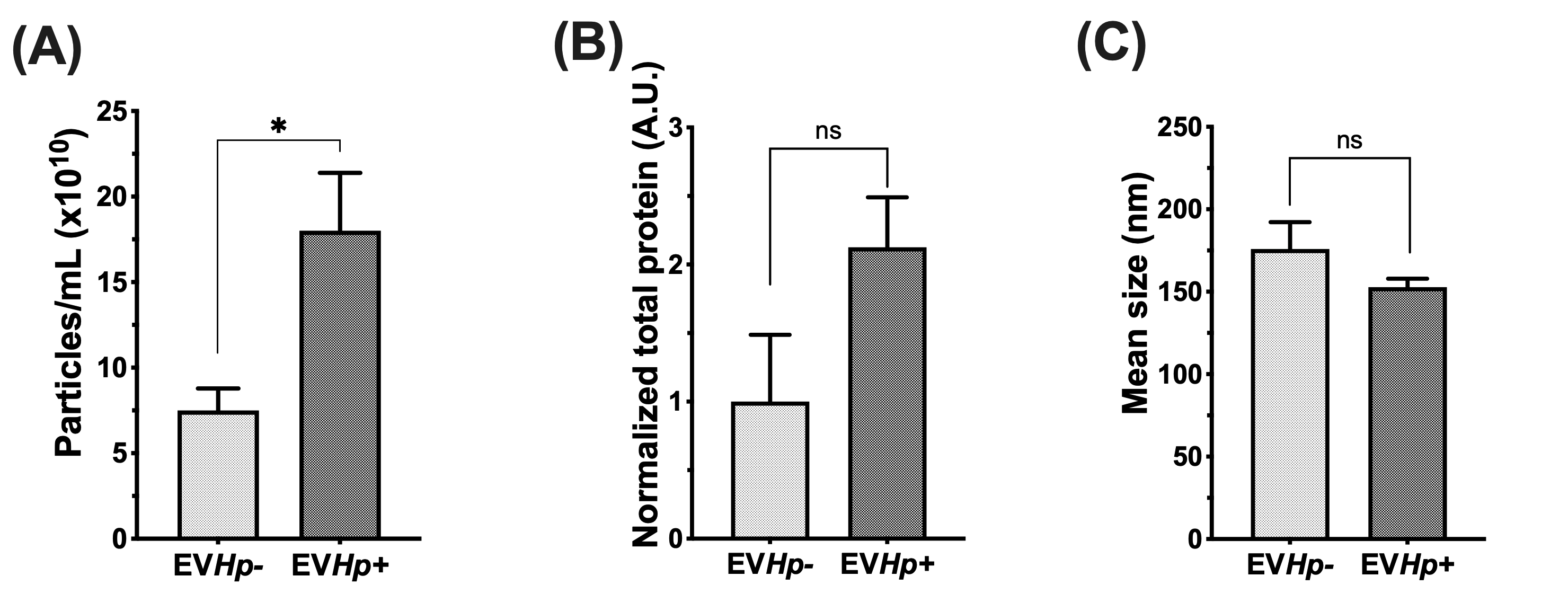


Supplementary Figure 1. EVs from GES-1 cells infected or not with H. pylori (EVHp+ and EVHp-, respectively) isolated using the Exo-spin kit. EVs were isolated from conditioned medium and characterized by NTA. (A) particle concentration; (B) normalized total protein; (C) mean size of EVs. Values shown were averaged from three independent experiments. A statistically significant difference (p value less than 0.05) is indicated as *; ns, not significant.

Supplementary Figure 2. Expression of cytokines in gastric cancer AGS cells stimulated with EVHp- or EVHp+ for 24 hours. Relative expression levels of (A) IL-8 ; (B) IL-6; (C) IL-10; (D) IL-1β and (E) IL-23. Values shown were averaged from four independent experiments.

**Supplementary Figure 3**. Incubation of GES-1 cells with EVHp+ increases the release of IL-23. The conditioned medium of GES-1 incubated with EVHp-, EVHp+, or without treatment (NT) for 24 hours were used to measure the levels of IL-23 by ELISA kit (Human IL-23 DuoSet ELISA, R&D Systems, Catalog number: DY1290-05). A statistically significant difference (p value less than 0.05) is indicated as *; ns, not significant. Values shown were averaged from three independent experiments.

Supplementary Figure 4. EVs isolated from GES-1 infected with *H. pylori* had a tendency to increase cell migration and invasion of gastric cancer Hs746T cells. Hs746T cells were left without stimulation (NT: no-treatment) and stimulated with EVHp- and EVHp+ for 24 h. Then, cell migration and invasion were determined in Transwell and Matrigel assays, respectively. (A) Hs746T cell migration averaged from thre independent experiments. Representative images are shown for Hs746T cells that migrated without stimulation (B), following stimulation with EVHp- (C) or EVHp+ (D); (E) Hs746T cell invasion averaged from three independent experiments. Representative images are shown for Hs746T cells that migrated without stimulation (F), following stimulation with EVHp- (G) or EVHp+ (H); Statistically significant differences (p values less than 0.01 and 0.0001) are indicated as ** or ****, respectively; ns, not significant.


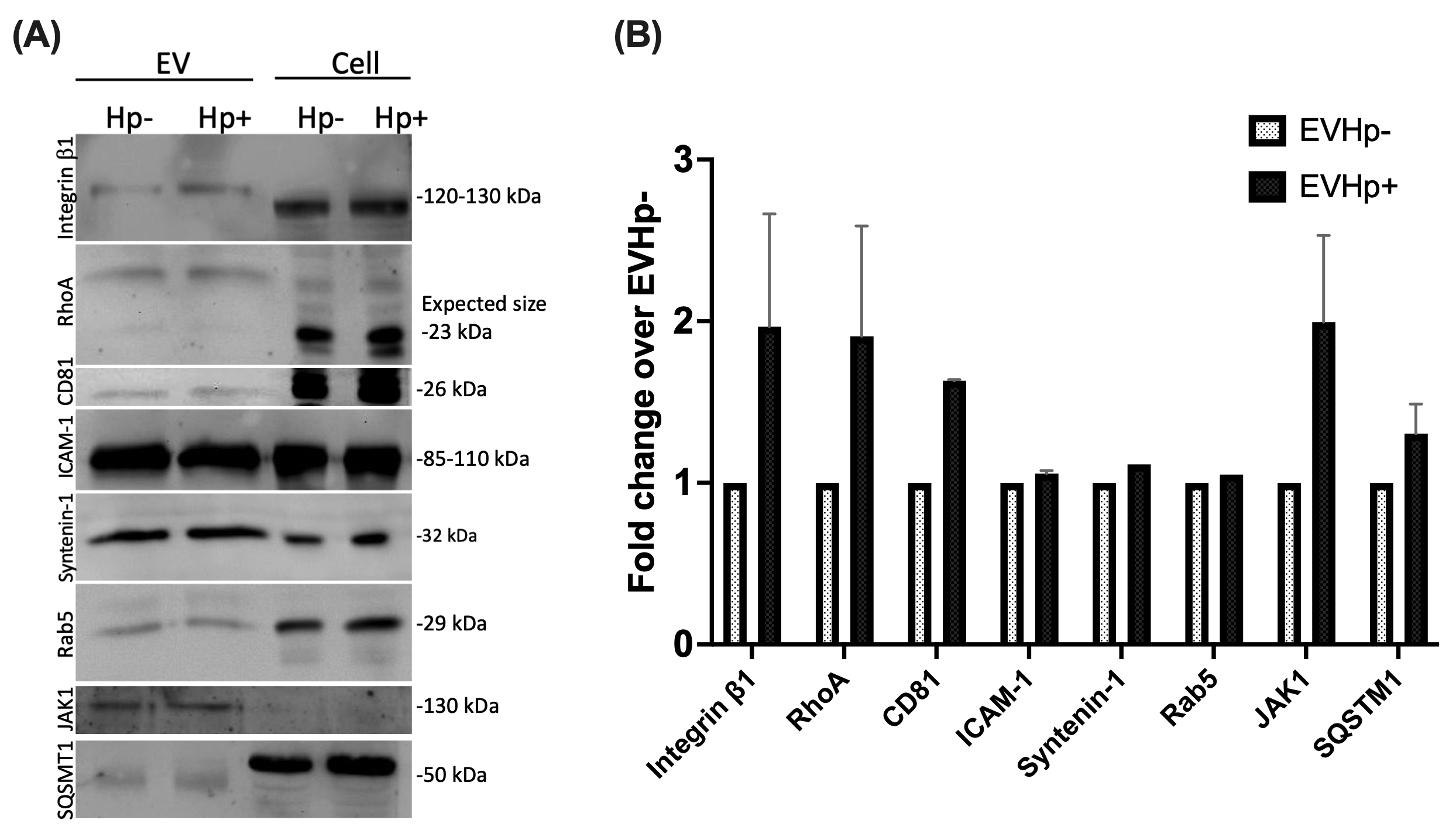


Supplementary Figure 5. (A) Representative Western blot and (B) the fold change in values obtained by scanning densitometric analysis of protein bands present in EVs functionally related to exosome biogenesis (CD81, Rab5), biological adhesion (ICAM-1, Integrin β1 and Syntenin-1), cell proliferation and metastasis (RhoA). JAK1 and SQSMT1 proteins that were only present in EVHp+ according to MS analysis, were also evaluated in EV samples. EV: Extracellular vesicle lysate; Cell: cell lysate; Hp-: not infected by H. pylori; Hp+: infected by H. pylori. WB was performed twice for Integrin β1, RhoA, CD81, ICAM-1, JAK1 and SQSMT1 and once for Syntenin-1 and Rab5

Supplementary Table 1. Primer sequences and annealing temperatures for qPCR

| Target gen | Sequence | Annealing temperature |
| --- | --- | --- |
| RNA 18S | 5´-GTGGAGCGATTTGTCTGGTT-3´ | 55 °C |
|  | 5´-CGCTGAGCCAGTCAGTGTAG-3´ |  |
| TNF-a | 5´-TCCGTGAAAACGGAGCTGAA-3´ | 58 °C |
|  | 5´-AGGCTCAGCAATGAGTGACA-3´ |  |
| IL-8 | 5´-TCTGGACCCCAAGGAAAACT-3´ | 58 °C |
|  | 5´-TTGCATCTGGCAACCCTACA-3´ |  |
| IL-6 | 5´-TGACCCAACCACAAATGCCA-3´ | 60 °C |
|  | 5´-AATCTGAGGTGCCCATGCTA-3´ |  |
| IL-33 | 5´AACACTCTGTGGAGCTCCAT-3´ | 60 °C |
|  | 5´AACACTCCAGGATCAGTCTTGC-3´ |  |
| IL-10 | 5´-TGGGTTGCCAAGCCTTGTCT-3´ | 60 °C |
|  | 5´-TCCACGGCCTTGCTCTTGTT-3´ |  |
| IL-1b | 5´-ACGGCTATAGCCTGGACTTT -3´ | 53 °C |
|  | 5´-TTTCTGTCAGGCGGGCTTTA-3´ |  |
| IL-23 | 5´-TGCTAGGATCGGATATTTTCACAGG-3´ | 53 °C |
|  | 5´-GAGGCTTGGAATCTGCTGAGTC-3´ |  |

Supplementary Table 2. Proteins found exclusively in EVHp+ compared to EVHp-

| 40S ribosomal protein S23;RPS23 |
| --- |
| 40S ribosomal protein S27;RPS27 |
| 40S ribosomal protein S28;RPS28 |
| 60 kDa heat shock protein, mitochondrial;HSPD1 |
| 60S acidic ribosomal protein P0;RPLP0 |
| 60S ribosomal protein L21;RPL21 |
| 60S ribosomal protein L23;RPL23 |
| 60S ribosomal protein L35a;RPL35A |
| 60S ribosomal protein L8;RPL8 |
| 60S ribosomal protein L9;RPL9P8 |
| Aminoacyl tRNA synthase complex-interacting multifunctional protein 2;AIMP2 |
| Amyloid-beta precursor protein;APP |
| Bifunctional glutamate/proline--tRNA ligase;EPRS1 |
| Cathepsin D;CTSD |
| Cysteine-rich motor neuron 1 protein;CRIM1 |
| D-3-phosphoglycerate dehydrogenase;PHGDH |
| Elongation factor Tu, mitochondrial;TUFM |
| Elongin-C;ELOC |
| Endophilin-A2;SH3GL1 |
| Eukaryotic initiation factor 4A-III;EIF4A3 |
| Eukaryotic translation initiation factor 2 subunit 2;EIF2S2 |
| F-actin-capping protein subunit beta;CAPZB |
| Fatty acid-binding protein 5;FABP5 |
| Flotillin-1;FLOT1 |
| Gap junction gamma-1 protein;GJC1 |
| Heterogeneous nuclear ribonucleoprotein A1-like 2;HNRNPA1L2 |
| Heterogeneous nuclear ribonucleoprotein A3;HNRNPA3 |
| Heterogeneous nuclear ribonucleoprotein L;HNRNPL |
| Heterogeneous nuclear ribonucleoprotein R;HNRNPR |
| Integral membrane protein 2B;ITM2B |
| Interleukin-6 receptor subunit beta;IL6ST |
| Keratinocyte-associated transmembrane protein 2;KCT2 |
| Kinesin-1 heavy chain;KIF5B |
| Lysine--tRNA ligase;KARS1 |
| Mast/stem cell growth factor receptor Kit;KIT |
| Neprilysin;MME |
| Non-POU domain-containing octamer-binding protein;NONO |
| Pleckstrin homology domain-containing family B member 2;PLEKHB2 |
| Pre-mRNA-processing factor 19;PRPF19 |
| Proteinase-activated receptor 1;F2R |
| Receptor-type tyrosine-protein phosphatase F;PTPRF |
| Ribonucleoside-diphosphate reductase large subunit;RRM1 |
| Sequestosome-1;SQSTM1 |
| Signal recognition particle subunit SRP72;SRP72 |
| Skin-specific protein 32;XP32 |
| Solute carrier family 43 member 3;SLC43A3 |
| Splicing factor U2AF 65 kDa subunit;U2AF2 |
| Staphylococcal nuclease domain-containing protein 1;SND1 |
| Transketolase;TKT |
| Transmembrane protein 59;TMEM59 |
| Tumor necrosis factor receptor superfamily member 10B;TNFRSF10B |
| Tyrosine-protein kinase JAK1;JAK1 |
| Vacuolar protein sorting-associated protein 35;VPS35 |

Supplementary Table 3. Proteins found exclusively in EVHp- compared to EVHp+

| Activated RNA polymerase II transcriptional coactivator p15;SUB1 |
| --- |
| Aldehyde dehydrogenase family 16 member A1;ALDH16A1 |
| Alpha-1-antichymotrypsin;SERPINA3 |
| Basal cell adhesion molecule;BCAM |
| Brain-specific angiogenesis inhibitor 1-associated protein 2;BAIAP2 |
| Ceruloplasmin;CP |
| Complement factor B;CFB |
| Kinesin-like protein KIF13B;KIF13B |
| Neuropilin-1;NRP1 |
| Palmitoyltransferase ZDHHC5;ZDHHC5 |
| Plasma kallikrein;KLKB1 |
| Protein FAM234A;FAM234A |
| Semaphorin-3F;SEMA3F |
| Single-stranded DNA-binding protein, mitochondrial;SSBP1 |
| Syndecan-2;SDC2 |
| Tetraspanin-9;TSPAN9 |
| Transthyretin;TTR |
| Y-box-binding protein 1;YBX1 |

Supplementary Table 4. Proteins enriched in EVHp+ compared to EVHp-

| 14-3-3 protein beta/alpha;YWHAB |
| --- |
| 14-3-3 protein epsilon;YWHAE |
| 14-3-3 protein sigma;SFN |
| 14-3-3 protein theta;YWHAQ |
| 14-3-3 protein zeta/delta;YWHAZ |
| 26S proteasome non-ATPase regulatory subunit 1;PSMD1 |
| 26S proteasome non-ATPase regulatory subunit 11;PSMD11 |
| 26S proteasome non-ATPase regulatory subunit 12;PSMD12 |
| 26S proteasome non-ATPase regulatory subunit 13;PSMD13 |
| 26S proteasome non-ATPase regulatory subunit 14;PSMD14 |
| 26S proteasome non-ATPase regulatory subunit 2;PSMD2 |
| 26S proteasome non-ATPase regulatory subunit 3;PSMD3 |
| 26S proteasome non-ATPase regulatory subunit 7;PSMD7 |
| 26S proteasome non-ATPase regulatory subunit 8;PSMD8 |
| 26S proteasome regulatory subunit 10B;PSMC6 |
| 26S proteasome regulatory subunit 4;PSMC1 |
| 26S proteasome regulatory subunit 6A;PSMC3 |
| 26S proteasome regulatory subunit 6B;PSMC4 |
| 26S proteasome regulatory subunit 7;PSMC2 |
| 26S proteasome regulatory subunit 8;PSMC5 |
| 40S ribosomal protein S11;RPS11 |
| 40S ribosomal protein S12;RPS12 |
| 40S ribosomal protein S13;RPS13 |
| 40S ribosomal protein S15a;RPS15A |
| 40S ribosomal protein S16;RPS16 |
| 40S ribosomal protein S18;RPS18 |
| 40S ribosomal protein S2;RPS2 |
| 40S ribosomal protein S20;RPS20 |
| 40S ribosomal protein S24;RPS24 |
| 40S ribosomal protein S25;RPS25 |
| 40S ribosomal protein S3;RPS3 |
| 40S ribosomal protein S3a;RPS3A |
| 40S ribosomal protein S4, X isoform;RPS4X |
| 40S ribosomal protein S4, Y isoform 1;RPS4Y1 |
| 40S ribosomal protein S6;RPS6 |
| 40S ribosomal protein S7;RPS7 |
| 40S ribosomal protein S8;RPS8 |
| 40S ribosomal protein S9;RPS9 |
| 40S ribosomal protein SA;RPSA |
| 4F2 cell-surface antigen heavy chain;SLC3A2 |
| 5'-3' exonuclease PLD3;PLD3 |
| 60S acidic ribosomal protein P0-like;RPLP0P6 |
| 60S acidic ribosomal protein P2;RPLP2 |
| 60S ribosomal protein L10-like;RPL10L |
| 60S ribosomal protein L10;RPL10 |
| 60S ribosomal protein L10a;RPL10A |
| 60S ribosomal protein L12;RPL12 |
| 60S ribosomal protein L13;RPL13 |
| 60S ribosomal protein L14;RPL14 |
| 60S ribosomal protein L15;RPL15 |
| 60S ribosomal protein L17;RPL17 |
| 60S ribosomal protein L18;RPL18 |
| 60S ribosomal protein L18a;RPL18A |
| 60S ribosomal protein L24;RPL24 |
| 60S ribosomal protein L3;RPL3 |
| 60S ribosomal protein L30;RPL30 |
| 60S ribosomal protein L32;RPL32 |
| 60S ribosomal protein L4;RPL4 |
| 60S ribosomal protein L5;RPL5 |
| 60S ribosomal protein L6;RPL6 |
| 60S ribosomal protein L7;RPL7 |
| 60S ribosomal protein L7a;RPL7A |
| Actin-related protein 3;ACTR3 |
| Actin, aortic smooth muscle;ACTA2 |
| Actin, cytoplasmic 1;ACTB |
| Acyl-CoA-binding protein;DBI |
| Adenine phosphoribosyltransferase;APRT |
| Adenylyl cyclase-associated protein 1;CAP1 |
| Adhesion G protein-coupled receptor E5;ADGRE5 |
| ADP-ribosylation factor 1;ARF1 |
| Agrin;AGRN |
| Alanine--tRNA ligase, cytoplasmic;AARS1 |
| Aldo-keto reductase family 1 member B1;AKR1B1 |
| Alpha-2-HS-glycoprotein;AHSG |
| Alpha-actinin-1;ACTN1 |
| Alpha-actinin-2;ACTN2 |
| Alpha-actinin-4;ACTN4 |
| Alpha-enolase;ENO1 |
| Alpha-fetoprotein;AFP |
| Alpha-soluble NSF attachment protein;NAPA |
| Annexin A1;ANXA1 |
| Annexin A11;ANXA11 |
| Annexin A2;ANXA2 |
| Annexin A4;ANXA4 |
| Annexin A5;ANXA5 |
| Annexin A6;ANXA6 |
| Annexin A7;ANXA7 |
| Antithrombin-III;SERPINC1 |
| AP-2 complex subunit beta;AP2B1 |
| AP-2 complex subunit mu;AP2M1 |
| Apolipoprotein A-IV;APOA4 |
| Apolipoprotein B-100;APOB |
| Apolipoprotein E;APOE |
| Arginine--tRNA ligase, cytoplasmic;RARS1 |
| Arrestin domain-containing protein 1;ARRDC1 |
| Aspartate--tRNA ligase, cytoplasmic;DARS1 |
| ATP-binding cassette sub-family E member 1;ABCE1 |
| ATP-citrate synthase;ACLY |
| ATP-dependent 6-phosphofructokinase, platelet type;PFKP |
| ATP-dependent RNA helicase A;DHX9 |
| Basement membrane-specific heparan sulfate proteoglycan core protein;HSPG2 |
| Basigin;BSG |
| Beta-2-glycoprotein 1;APOH |
| Beta-actin-like protein 2;ACTBL2 |
| Beta-enolase;ENO3 |
| Bifunctional purine biosynthesis protein ATIC;ATIC |
| Brain acid soluble protein 1;BASP1 |
| BRO1 domain-containing protein BROX;BROX |
| C-1-tetrahydrofolate synthase, cytoplasmic;MTHFD1 |
| CAD protein;CAD |
| Cadherin-13;CDH13 |
| Calpain-2 catalytic subunit;CAPN2 |
| Carbonyl reductase [NADPH] 1;CBR1 |
| Caspase-14;CASP14 |
| Cation-dependent mannose-6-phosphate receptor;M6PR |
| Cation-independent mannose-6-phosphate receptor;IGF2R |
| Caveolin-1;CAV1 |
| CCN family member 1;CCN1 |
| CD151 antigen;CD151 |
| CD166 antigen;ALCAM |
| CD276 antigen;CD276 |
| CD44 antigen;CD44 |
| CD59 glycoprotein;CD59 |
| CD63 antigen;CD63 |
| CD70 antigen;CD70 |
| CD81 antigen;CD81 |
| CD82 antigen;CD82 |
| CD9 antigen;CD9 |
| Cell cycle control protein 50A;TMEM30A |
| Cell division control protein 42 homolog;CDC42 |
| Cell surface glycoprotein MUC18;MCAM |
| Charged multivesicular body protein 1a;CHMP1A |
| Charged multivesicular body protein 1b;CHMP1B |
| Charged multivesicular body protein 2a;CHMP2A |
| Charged multivesicular body protein 2b;CHMP2B |
| Charged multivesicular body protein 4a;CHMP4A |
| Charged multivesicular body protein 4b;CHMP4B |
| Charged multivesicular body protein 5;CHMP5 |
| Chondroitin sulfate proteoglycan 4;CSPG4 |
| Clathrin heavy chain 1;CLTC |
| Clathrin heavy chain 2;CLTCL1 |
| Claudin-11;CLDN11 |
| Clusterin;CLU |
| Coatomer subunit alpha;COPA |
| Cofilin-1;CFL1 |
| Collagen alpha-1(XVIII) chain;COL18A1 |
| Collagen alpha-2(IV) chain;COL4A2 |
| Complement component C9;C9 |
| Complement decay-accelerating factor;CD55 |
| Copine-1;CPNE1 |
| Copine-2;CPNE2 |
| Copine-3;CPNE3 |
| Core histone macro-H2A.1;MACROH2A1 |
| Core histone macro-H2A.2;MACROH2A2 |
| Coronin-1C;CORO1C |
| Cullin-4B;CUL4B |
| Cytoplasmic dynein 1 heavy chain 1;DYNC1H1 |
| Cytoplasmic FMR1-interacting protein 2;CYFIP2 |
| Deoxyribose-phosphate aldolase;DERA |
| Dermcidin;DCD |
| Desmocollin-1;DSC1 |
| Desmoglein-1;DSG1 |
| Desmoplakin;DSP |
| Destrin;DSTN |
| Disintegrin and metalloproteinase domain-containing protein 10;ADAM10 |
| Disintegrin and metalloproteinase domain-containing protein 9;ADAM9 |
| DNA topoisomerase 2-alpha;TOP2A |
| DNA-dependent protein kinase catalytic subunit;PRKDC |
| DnaJ homolog subfamily A member 1;DNAJA1 |
| DnaJ homolog subfamily A member 2;DNAJA2 |
| EGF-like repeat and discoidin I-like domain-containing protein 3;EDIL3 |
| EH domain-containing protein 1;EHD1 |
| EH domain-containing protein 4;EHD4 |
| Elongation factor 1-gamma;EEF1G |
| Elongation factor 2;EEF2 |
| Endoglin;ENG |
| Endoplasmic reticulum chaperone BiP;HSPA5 |
| Endoplasmin;HSP90B1 |
| Endothelin-converting enzyme 1;ECE1 |
| Ephrin type-A receptor 2;EPHA2 |
| Ephrin type-B receptor 1;EPHB1 |
| Epidermal growth factor receptor;EGFR |
| Equilibrative nucleoside transporter 1;SLC29A1 |
| Erythrocyte band 7 integral membrane protein;STOM |
| Eukaryotic initiation factor 4A-I;EIF4A1 |
| Eukaryotic peptide chain release factor GTP-binding subunit ERF3A;GSPT1 |
| Eukaryotic translation initiation factor 2 subunit 1;EIF2S1 |
| Eukaryotic translation initiation factor 2 subunit 3;EIF2S3 |
| Eukaryotic translation initiation factor 3 subunit A;EIF3A |
| Eukaryotic translation initiation factor 3 subunit B;EIF3B |
| Eukaryotic translation initiation factor 3 subunit C-like protein;EIF3CL |
| Eukaryotic translation initiation factor 3 subunit F;EIF3F |
| Eukaryotic translation initiation factor 3 subunit L;EIF3L |
| Eukaryotic translation initiation factor 5A-2;EIF5A2 |
| Exportin-1;XPO1 |
| Exportin-2;CSE1L |
| Ezrin;EZR |
| F-actin-capping protein subunit alpha-1;CAPZA1 |
| FACT complex subunit SSRP1;SSRP1 |
| Fascin;FSCN1 |
| Fatty acid synthase;FASN |
| Ferritin light chain;FTL |
| Fibrinogen alpha chain;FGA |
| Filaggrin-2;FLG2 |
| Filamin-B;FLNB |
| Filamin-C;FLNC |
| Frizzled-2;FZD2 |
| Fructose-bisphosphate aldolase A;ALDOA |
| Fructose-bisphosphate aldolase C;ALDOC |
| Galectin-1;LGALS1 |
| Galectin-3-binding protein;LGALS3BP |
| Glucose-6-phosphate 1-dehydrogenase;G6PD |
| Glucose-6-phosphate isomerase;GPI |
| Glutathione S-transferase P;GSTP1 |
| Glyceraldehyde-3-phosphate dehydrogenase;GAPDH |
| Glycine--tRNA ligase;GARS1 |
| Glypican-1;GPC1 |
| Golgi-associated plant pathogenesis-related protein 1;GLIPR2 |
| Golgin subfamily A member 7;GOLGA7 |
| Gremlin-1;GREM1 |
| GTP-binding nuclear protein Ran;RAN |
| GTPase HRas;HRAS |
| Guanine nucleotide-binding protein G(i) subunit alpha-2;GNAI2 |
| Guanine nucleotide-binding protein G(i) subunit alpha;GNAI3 |
| Guanine nucleotide-binding protein G(I)/G(S)/G(O) subunit gamma-12;GNG12 |
| Guanine nucleotide-binding protein G(I)/G(S)/G(O) subunit gamma-5;GNG5 |
| Guanine nucleotide-binding protein G(I)/G(S)/G(T) subunit beta-1;GNB1 |
| Guanine nucleotide-binding protein G(I)/G(S)/G(T) subunit beta-2;GNB2 |
| Guanine nucleotide-binding protein G(I)/G(S)/G(T) subunit beta-3;GNB3 |
| Guanine nucleotide-binding protein G(s) subunit alpha isoforms XLas;GNAS |
| Guanine nucleotide-binding protein G(t) subunit alpha-3;GNAT3 |
| Heat shock 70 kDa protein 1-like;HSPA1L |
| Heat shock 70 kDa protein 1A;HSPA1A |
| Heat shock 70 kDa protein 4;HSPA4 |
| Heat shock cognate 71 kDa protein;HSPA8 |
| Heat shock protein 105 kDa;HSPH1 |
| Heat shock protein beta-1;HSPB1 |
| Heat shock protein HSP 90-alpha;HSP90AA1 |
| Heat shock protein HSP 90-beta;HSP90AB1 |
| Heparin cofactor 2;SERPIND1 |
| Heterogeneous nuclear ribonucleoprotein D0;HNRNPD |
| Heterogeneous nuclear ribonucleoprotein H2;HNRNPH2 |
| Heterogeneous nuclear ribonucleoprotein K;HNRNPK |
| Heterogeneous nuclear ribonucleoprotein U;HNRNPU |
| High affinity cationic amino acid transporter 1;SLC7A1 |
| Histone H1.0;H1-0 |
| Histone H1.3;H1-3 |
| Histone H1.5;H1-5 |
| Histone H2A type 1-A;H2AC1 |
| Histone H2A type 1-J;H2AC14 |
| Histone H2A.Z;H2AZ1 |
| Histone H2B type 1-N;H2BC15 |
| Histone H2B type 1-O;H2BC17 |
| Histone H3.1t;H3-4 |
| HLA class I histocompatibility antigen, A alpha chain;HLA-A |
| HLA class I histocompatibility antigen, B alpha chain;HLA-B |
| Hornerin;HRNR |
| Hsc70-interacting protein;ST13 |
| Hypoxanthine-guanine phosphoribosyltransferase;HPRT1 |
| Hypoxia up-regulated protein 1;HYOU1 |
| Immunoglobulin superfamily member 8;IGSF8 |
| Importin subunit alpha-1;KPNA2 |
| Importin subunit beta-1;KPNB1 |
| Integrin alpha-2;ITGA2 |
| Integrin alpha-3;ITGA3 |
| Integrin alpha-5;ITGA5 |
| Integrin alpha-6;ITGA6 |
| Integrin alpha-V;ITGAV |
| Integrin beta-1;ITGB1 |
| Integrin beta-3;ITGB3 |
| Inter-alpha-trypsin inhibitor heavy chain H2;ITIH2 |
| Inter-alpha-trypsin inhibitor heavy chain H4;ITIH4 |
| Intercellular adhesion molecule 1;ICAM1 |
| Interferon-induced transmembrane protein 1;IFITM1 |
| Isocitrate dehydrogenase [NADP] cytoplasmic;IDH1 |
| Isoleucine--tRNA ligase, cytoplasmic;IARS1 |
| IST1 homolog;IST1 |
| Junction plakoglobin;JUP |
| Junctional adhesion molecule A;F11R |
| Keratinocyte proline-rich protein;KPRP |
| L-lactate dehydrogenase A chain;LDHA |
| L-lactate dehydrogenase B chain;LDHB |
| Lactadherin;MFGE8 |
| Laminin subunit alpha-5;LAMA5 |
| Laminin subunit beta-1;LAMB1 |
| Laminin subunit gamma-1;LAMC1 |
| Large neutral amino acids transporter small subunit 1;SLC7A5 |
| Leukocyte surface antigen CD47;CD47 |
| Low-density lipoprotein receptor;LDLR |
| Lysosome membrane protein 2;SCARB2 |
| Lysosome-associated membrane glycoprotein 1;LAMP1 |
| Lysosome-associated membrane glycoprotein 2;LAMP2 |
| Major vault protein;MVP |
| Malate dehydrogenase, mitochondrial;MDH2 |
| MARCKS-related protein;MARCKSL1 |
| Matrilin-2;MATN2 |
| Membrane cofactor protein;CD46 |
| Metal cation symporter ZIP14;SLC39A14 |
| MIT domain-containing protein 1;MITD1 |
| MOB kinase activator 1A;MOB1A |
| Moesin;MSN |
| Monocarboxylate transporter 1;SLC16A1 |
| Monocarboxylate transporter 4;SLC16A3 |
| Multifunctional protein ADE2;PAICS |
| Multivesicular body subunit 12A;MVB12A |
| Myelin protein zero-like protein 1;MPZL1 |
| Myoferlin;MYOF |
| Myosin light polypeptide 6;MYL6 |
| Myristoylated alanine-rich C-kinase substrate;MARCKS |
| Na(+)/H(+) exchange regulatory cofactor NHE-RF1;SLC9A3R1 |
| Na(+)/H(+) exchange regulatory cofactor NHE-RF2;SLC9A3R2 |
| Nectin-2;NECTIN2 |
| Neural cell adhesion molecule L1;L1CAM |
| Neuroblast differentiation-associated protein AHNAK;AHNAK |
| Neurogenic locus notch homolog protein 1;NOTCH1 |
| Neurogenic locus notch homolog protein 2;NOTCH2 |
| Neuroplastin;NPTN |
| Neutral alpha-glucosidase AB;GANAB |
| Neutral amino acid transporter B(0);SLC1A5 |
| Nicastrin;NCSTN |
| Nidogen-1;NID1 |
| Nucleolar protein 56;NOP56 |
| Nucleophosmin;NPM1 |
| Nucleoside diphosphate kinase B;NME2 |
| Nucleosome assembly protein 1-like 1;NAP1L1 |
| Pentraxin-related protein PTX3;PTX3 |
| Peptidyl-prolyl cis-trans isomerase A;PPIA |
| Peptidyl-prolyl cis-trans isomerase FKBP4;FKBP4 |
| Periostin;POSTN |
| Peroxiredoxin-1;PRDX1 |
| Peroxiredoxin-2;PRDX2 |
| Peroxiredoxin-6;PRDX6 |
| Phosphoglycerate kinase 1;PGK1 |
| Phosphoglycerate mutase 1;PGAM1 |
| Phospholipid scramblase 1;PLSCR1 |
| Pigment epithelium-derived factor;SERPINF1 |
| Pituitary tumor-transforming gene 1 protein-interacting protein;PTTG1IP |
| Plasma membrane calcium-transporting ATPase 3;ATP2B3 |
| Plastin-3;PLS3 |
| Plexin-B2;PLXNB2 |
| Podocalyxin;PODXL |
| Poliovirus receptor;PVR |
| Poly(rC)-binding protein 2;PCBP2 |
| Potassium-transporting ATPase alpha chain 2;ATP12A |
| Pre-mRNA-splicing factor ATP-dependent RNA helicase DHX15;DHX15 |
| Probable ATP-dependent RNA helicase DDX17;DDX17 |
| Profilin-1;PFN1 |
| Programmed cell death 6-interacting protein;PDCD6IP |
| Programmed cell death protein 6;PDCD6 |
| Progranulin;GRN |
| Proliferating cell nuclear antigen;PCNA |
| Proliferation-associated protein 2G4;PA2G4 |
| Prostaglandin F2 receptor negative regulator;PTGFRN |
| Proteasome activator complex subunit 3;PSME3 |
| Proteasome subunit alpha type-1;PSMA1 |
| Proteasome subunit alpha type-2;PSMA2 |
| Proteasome subunit alpha type-3;PSMA3 |
| Proteasome subunit alpha type-4;PSMA4 |
| Proteasome subunit alpha type-5;PSMA5 |
| Proteasome subunit alpha type-6;PSMA6 |
| Proteasome subunit alpha type-7;PSMA7 |
| Proteasome subunit beta type-1;PSMB1 |
| Proteasome subunit beta type-2;PSMB2 |
| Proteasome subunit beta type-3;PSMB3 |
| Proteasome subunit beta type-4;PSMB4 |
| Proteasome subunit beta type-5;PSMB5 |
| Proteasome subunit beta type-6;PSMB6 |
| Proteasome subunit beta type-7;PSMB7 |
| Protein AMBP;AMBP |
| Protein DEK;DEK |
| Protein kinase C and casein kinase substrate in neurons protein 3;PACSIN3 |
| Protein Niban 2;NIBAN2 |
| Protein S100-A10;S100A10 |
| Protein S100-A11;S100A11 |
| Protein S100-A16;S100A16 |
| Protein S100-A6;S100A6 |
| Protein S100-A7;S100A7 |
| Protein XRP2;RP2 |
| Proteolipid protein 2;PLP2 |
| Putative 40S ribosomal protein S26-like 1;RPS26P11 |
| Putative elongation factor 1-alpha-like 3;EEF1A1P5 |
| Putative heat shock 70 kDa protein 7;HSPA7 |
| Putative HLA class I histocompatibility antigen, alpha chain H;HLA-H |
| Pyruvate kinase PKM;PKM |
| Ras GTPase-activating-like protein IQGAP1;IQGAP1 |
| Ras-related C3 botulinum toxin substrate 1;RAC1 |
| Ras-related C3 botulinum toxin substrate 3;RAC3 |
| Ras-related protein R-Ras2;RRAS2 |
| Ras-related protein Rab-10;RAB10 |
| Ras-related protein Rab-11B;RAB11B |
| Ras-related protein Rab-13;RAB13 |
| Ras-related protein Rab-14;RAB14 |
| Ras-related protein Rab-1A;RAB1A |
| Ras-related protein Rab-1B;RAB1B |
| Ras-related protein Rab-21;RAB21 |
| Ras-related protein Rab-2B;RAB2B |
| Ras-related protein Rab-35;RAB35 |
| Ras-related protein Rab-5A;RAB5A |
| Ras-related protein Rab-5B;RAB5B |
| Ras-related protein Rab-5C;RAB5C |
| Ras-related protein Rab-7a;RAB7A |
| Ras-related protein Rab-8A;RAB8A |
| Ras-related protein Rab-8B;RAB8B |
| Ras-related protein Ral-A;RALA |
| Ras-related protein Ral-B;RALB |
| Ras-related protein Rap-1b-like protein;- |
| Ras-related protein Rap-1b;RAP1B |
| Ras-related protein Rap-2b;RAP2B |
| Receptor of activated protein C kinase 1;RACK1 |
| Reticulon-4;RTN4 |
| Retinoic acid-induced protein 3;GPRC5A |
| Rho-associated protein kinase 2;ROCK2 |
| Ribonuclease inhibitor;RNH1 |
| Ribose-phosphate pyrophosphokinase 1;PRPS1 |
| Ribosomal L1 domain-containing protein 1;RSL1D1 |
| RuvB-like 1;RUVBL1 |
| RuvB-like 2;RUVBL2 |
| Scavenger receptor class B member 1;SCARB1 |
| Secretory carrier-associated membrane protein 3;SCAMP3 |
| Serine incorporator 1;SERINC1 |
| Serine/arginine-rich splicing factor 1;SRSF1 |
| Serine/arginine-rich splicing factor 3;SRSF3 |
| Serine/arginine-rich splicing factor 7;SRSF7 |
| Serine/threonine-protein phosphatase 2A 65 kDa regulatory subunit A alpha isoform;PPP2R1A |
| Serine/threonine-protein phosphatase PP1-beta catalytic subunit;PPP1CB |
| Small nuclear ribonucleoprotein Sm D3;SNRPD3 |
| Small nuclear ribonucleoprotein-associated proteins B and B';SNRPB |
| Sodium-coupled neutral amino acid transporter 2;SLC38A2 |
| Sodium/potassium-transporting ATPase subunit alpha-1;ATP1A1 |
| Sodium/potassium-transporting ATPase subunit beta-1;ATP1B1 |
| Sodium/potassium-transporting ATPase subunit beta-3;ATP1B3 |
| Solute carrier family 2, facilitated glucose transporter member 1;SLC2A1 |
| Sorcin;SRI |
| SPARC;SPARC |
| Splicing factor 3B subunit 1;SF3B1 |
| Splicing factor 3B subunit 3;SF3B3 |
| Splicing factor, proline- and glutamine-rich;SFPQ |
| Stress-70 protein, mitochondrial;HSPA9 |
| Stress-induced-phosphoprotein 1;STIP1 |
| Sulfhydryl oxidase 1;QSOX1 |
| Superoxide dismutase [Cu-Zn];SOD1 |
| Suprabasin;SBSN |
| Synaptic vesicle membrane protein VAT-1 homolog;VAT1 |
| Synaptobrevin homolog YKT6;YKT6 |
| Synaptosomal-associated protein 23;SNAP23 |
| Syndecan-4;SDC4 |
| Syntaxin-4;STX4 |
| Syntaxin-binding protein 3;STXBP3 |
| Syntenin-1;SDCBP |
| T-complex protein 1 subunit alpha;TCP1 |
| T-complex protein 1 subunit beta;CCT2 |
| T-complex protein 1 subunit delta;CCT4 |
| T-complex protein 1 subunit epsilon;CCT5 |
| T-complex protein 1 subunit eta;CCT7 |
| T-complex protein 1 subunit gamma;CCT3 |
| T-complex protein 1 subunit theta;CCT8 |
| T-complex protein 1 subunit zeta-2;CCT6B |
| T-complex protein 1 subunit zeta;CCT6A |
| Tenascin;TNC |
| Testican-1;SPOCK1 |
| Tetranectin;CLEC3B |
| Tetraspanin-14;TSPAN14 |
| Tetraspanin-3;TSPAN3 |
| Tetraspanin-6;TSPAN6 |
| Thioredoxin;TXN |
| Threonine--tRNA ligase 1, cytoplasmic;TARS1 |
| Thy-1 membrane glycoprotein;THY1 |
| Thyroxine-binding globulin;SERPINA7 |
| Tissue-type plasminogen activator;PLAT |
| Toll-interacting protein;TOLLIP |
| Transaldolase;TALDO1 |
| Transferrin receptor protein 1;TFRC |
| Transformer-2 protein homolog alpha;TRA2A |
| Transforming growth factor-beta-induced protein ig-h3;TGFBI |
| Transforming protein RhoA;RHOA |
| Transitional endoplasmic reticulum ATPase;VCP |
| Trophoblast glycoprotein;TPBG |
| Tropomyosin alpha-3 chain;TPM3 |
| Tubulin beta chain;TUBB |
| Tubulin beta-2A chain;TUBB2A |
| Tubulin beta-4A chain;TUBB4A |
| Tubulin beta-6 chain;TUBB6 |
| Tubulointerstitial nephritis antigen-like;TINAGL1 |
| Tumor susceptibility gene 101 protein;TSG101 |
| Tyrosine-protein kinase receptor UFO;AXL |
| Ubiquitin-like modifier-activating enzyme 1;UBA1 |
| UDP-N-acetylhexosamine pyrophosphorylase;UAP1 |
| Unconventional myosin-Ic;MYO1C |
| Vacuolar protein sorting-associated protein 37B;VPS37B |
| Vacuolar protein sorting-associated protein 4A;VPS4A |
| Vacuolar protein sorting-associated protein VTA1 homolog;VTA1 |
| Vimentin;VIM |
| Vitamin D-binding protein;GC |
